# Divergent macrophage-regulated T cell states determine response to Bacillus Calmette-Guérin vaccine in high-risk bladder cancer

**DOI:** 10.1172/JCI200442

**Published:** 2026-04-21

**Authors:** Ryan J. Brown, Mairah T. Khan, Andrew J. Houston, Hongshen Niu, Joseph R. Podojil, Bonnie Choy, Weiguo Cui, Joshua J. Meeks

**Affiliations:** 1 Department of Microbiology and Immunology and; 2 Department of Pathology, Northwestern University Feinberg School of Medicine, Chicago, Illinois, USA.; 3 Department of Microbiology and Immunology, Medical College of Wisconsin, Milwaukee, Wisconsin, USA.; 4 Department of Urology and; 5 Department of Biochemistry and Molecular Genetics, Northwestern University Feinberg School of Medicine, Chicago, Illinois, USA.; 6 Jesse Brown Department of Veterans Affairs Medical Center, Chicago, Illinois, USA.

**Keywords:** Immunology, Oncology, Adaptive immunity, Tuberculosis, Urology

## Abstract

**BACKGROUND:**

Primary therapy for high-risk bladder cancer (BCa) is repeated instillations of the tuberculosis vaccine Bacillus Calmette-Guérin (BCG). Although BCG reduces the risk of recurrence by more than half, the mechanisms underlying its immune-activating effects remain unknown. Our objective was to investigate how the immune response differs between BCG responders and nonresponders and to compare systemic and local immune responses.

**METHODS:**

We performed scRNA-seq of isolated immune cells adjacent to high-risk bladders in BCG responders and nonresponders before and after BCG. We also compared concurrent scRNA-seq profiles of circulating immune cell populations with those of bladder immune cells.

**RESULTS:**

We observed an increase in Th17-like Th1 cells in BCG responders, characterized by greater expression of proinflammatory cytokines. By contrast, nonresponders showed increased CD8^+^ T cell exhaustion and Treg cells. We found that the primary mechanism driving divergent T cell activity is altered polarization and immunosuppressive signaling with myeloid cells. Using a machine learning–based approach, we identified that Th17-like Th1 cytokines, such as IL-17, IL-21, and IL-26, are predictive of response, which was subsequently validated in a separate BCG-treated BCa cohort.

**CONCLUSION:**

Together, these findings suggest that dynamic regulation of myeloid–T cell interactions can be critical for outcomes of BCG-treated BCa.

**FUNDING:**

BX005599 and BX003692 (Veterans Health Administration), HT94252410507 (Department of Defense), R01CA298333 (National Cancer Institute), and Robert H. Lurie Comprehensive Cancer Center H Foundation Core Facility Pilot Project Award.

## Abstract 2

From analysis of the tumor microenvironment in patients with bladder cancer, we identify that the dynamic regulation of myeloid-T cell interactions can be critical for outcomes of BCG.

## Graphical abstract

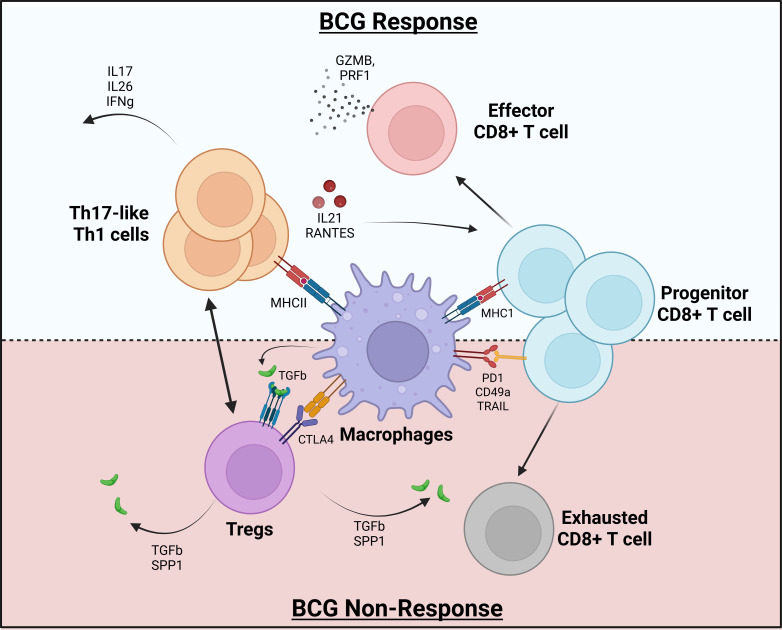

## Introduction

Bladder cancer (BCa) is the eighth most common cancer worldwide and the fourth most common among men (1–3). It is also the most economically burdensome malignancy, largely due to its high recurrence rate, which necessitates frequent and invasive monitoring procedures, such as cystoscopies to detect new or recurrent tumors (4). Most cases (~80%) are confined to the superficial lining of the bladder and are classified as non-muscle-invasive bladder cancer (NMIBC) (1, 3) Standard management of NMIBC involves surgical resection followed by adjuvant immunotherapy, but nearly one-third of tumors recur within 2 years (5). The most effective and widely used therapy is intravesical administration of *Mycobacterium bovis* Bacillus Calmette-Guérin (BCG), which has been shown to reduce recurrence by more than 50% (6). However, despite its clinical utility, approximately one-third of patients fail to respond to BCG (7–9). Among these BCG-unresponsive cases, 25%–30% progress to muscle-invasive disease, necessitating systemic therapy or radical cystectomy (10). Compounding these challenges, global manufacturing shortages have further constrained access to BCG, prompting rationing strategies in many regions (11).

A major limitation to developing new strategies to improve or replace BCG is the limited knowledge of the immune mechanisms by which BCG causes BCa eradication. BCG is an attenuated derivative of *Mycobacterium tuberculosis* (*Mtb*), generated through deletion of the RD1 locus encoding the ESX-1 type VII secretion system (12, 13). Originally developed as a vaccine to prevent *Mtb* infection, BCG was later found to exhibit therapeutic activity across multiple solid tumors (14). Extensive immune characterization of *Mtb* has identified several processes that may be critical to BCG activity. *Mtb* infects the alveolar macrophages of the lung, resulting in activation of the innate immune system and recruitment of Th1 CD4^+^ T cells that ultimately create granulomas and tissue necrosis (15). Conversely, *Mtb* dampens the immune response through induction of immunosuppressive cells and signaling, such as TGF-β and IL-10, and downregulation of MHC class II (MHCII) on antigen-presenting cells, and latent pulmonary tuberculosis has been associated with exhaustion in CD4^+^ and CD8^+^ T cells (16–18). Attempts to ameliorate exhaustion through PD1 immune checkpoint immunotherapy and T cell exhaustion in BCa remain controversial, but tumors with increased expression of PDL1 have been reported to have diminished response to BCG (19). Pembrolizumab was approved for BCG-unresponsive BCa in 2019, and a recent clinical trial combining anti-PD1 therapy with BCG prolongs recurrence-free survival of BCa (20, 21). Therefore, investigating the mechanisms of immune-mediated BCG could help identify how to synergize immune checkpoint blockade with BCG and discover new therapies for treating patients who are unresponsive or unable to receive adequate BCG.

To evaluate how the tumor microenvironment of the bladder evolves during BCG therapy, we performed single-cell transcriptome analysis of the adjacent normal bladder tissue and circulating immune cell populations from patients treated with BCG for high-risk BCa. Through cellular characterization, cell–cell interactions, and machine learning, we compared BCG-naive to BCG-responsive and nonresponsive bladders, identifying immune populations and gene expression programs that parallel changes found in latent *Mtb*. Specifically, we identify a Th17-like Th1 CD4^+^ T cell population that associates with clinical response to BCG. Interestingly, we found that BCG responders had a more activated macrophage profile with an increase in antigen presentation. Conversely, BCG nonresponder macrophages exhibited reduced MHCII presentation, increased TGF-β signaling, and enhanced coinhibitory pathways. In alignment with this, BCG-nonresponding patients had a corresponding increase in immunosuppressive Tregs and exhausted CD8^+^ T cells. Together, these results suggest that strategies applied to activate the immune system in *Mtb* and potentially block exhaustion could be leveraged to overcome BCG resistance in patients with BCa.

## Results

**Figure 1:**
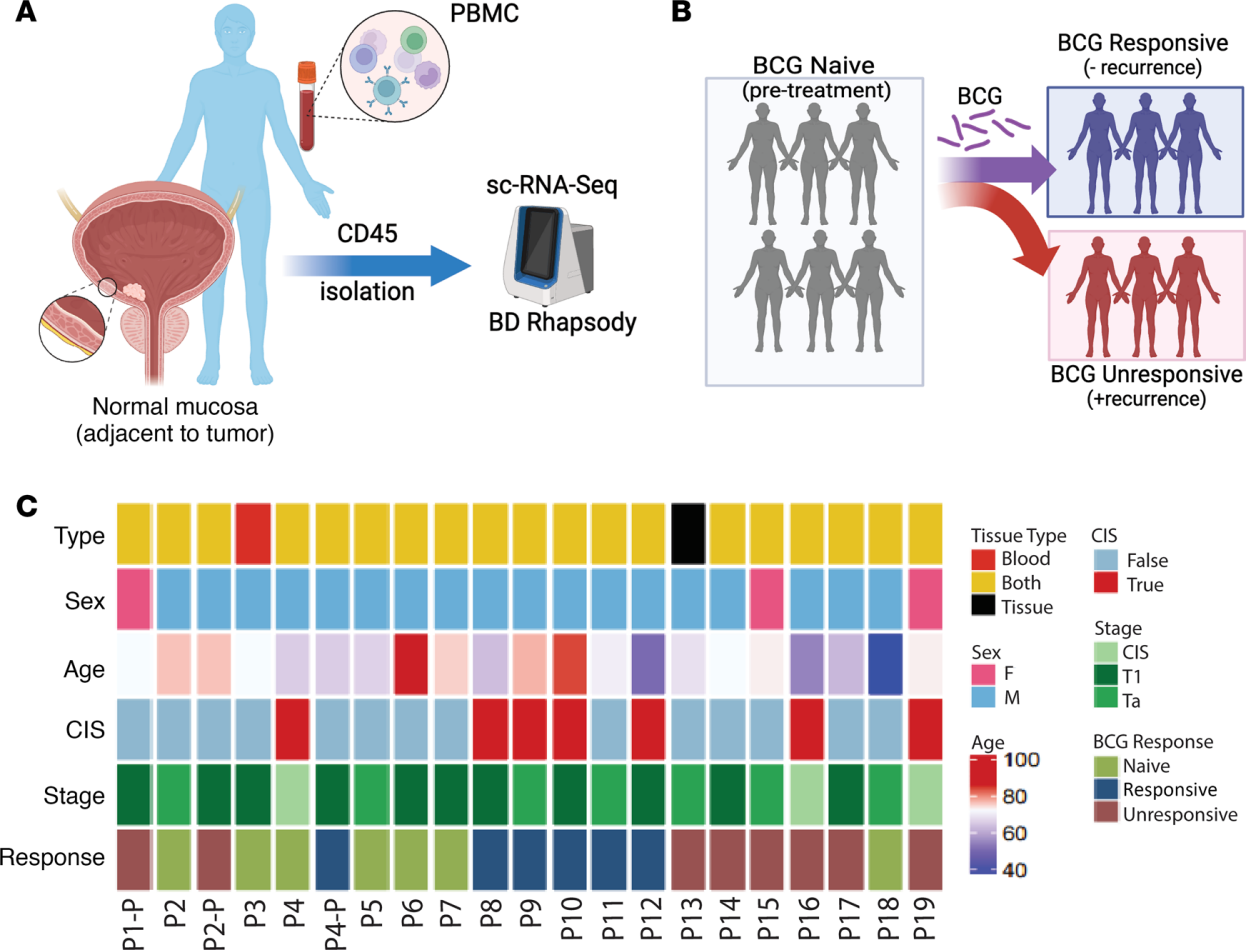
Study design and patient cohort for immune profiling of the BCa tumor microenvironment during BCG therapy. (**A**) Sample collection from patients with BCa. For most patients, whole and bladder biopsies adjacent to the tumor were collected prospectively with CD45^+^ isolation and analyzed by scRNA-seq using the BD Rhapsody system. (**B**) Patients were prospectively enrolled at 2 distinct time points: before (BCG naive) and after BCG therapy. After BCG therapy, patients were classified as either responsive or resistant, depending on the result of the bladder biopsy. (**C**) Metadata of each patient’s covariates were sequenced, including sex, age, tumor stage, tissue compartments, and the patient’s BCG response CIS, carcinoma in situ.

To investigate how the local and systemic immune systems evolve during intravesical BCG treatment for BCa, scRNA-seq was performed on blood and tissue biopsies from patients with high-risk NMIBC (Figure 1A). To avoid possible contribution and overrepresentation of epithelial cells from invasive BCa, tissue biopsies were taken adjacent to the tumor in visually “normal” areas of the bladder. CD45 enrichment was performed on the tissue samples during the processing before scRNA-seq (Figure 1A). To further elucidate the mechanism of an effective BCG response, data were collected from BCG-naive (*n* = 7), BCG-responsive (*n* = 6), and BCG-unresponsive (*n* = 8) patients among a total of 19 patients (Figure 1, B and C). In parallel, scRNA-seq was performed on PBMCs to augment the analysis of tissue specimens (Figure 1, A–C).

### Immune landscape of the bladder and circulation of BCG-treated patients.

**Figure 2:**
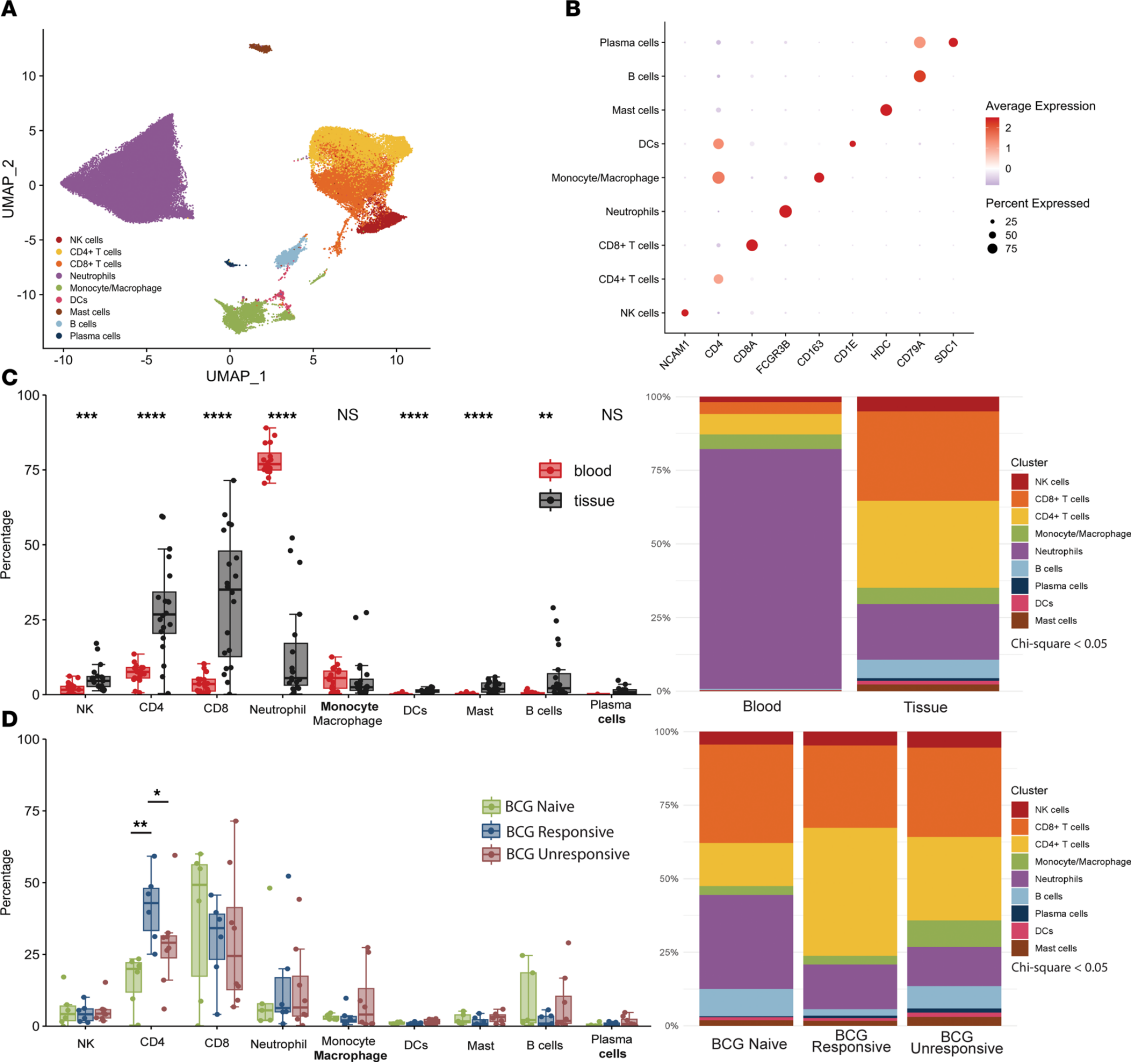
Comprehensive dissection and clustering of immune cells from BCG-treated bladders. (**A**) UMAP plot of all immune cells collected across 19 patients from the blood and bladder tissue of BCG-naive, -responsive, and -unresponsive groups. (**B**) Dot plot of representative genes expressed in each major cluster. Dot size represents percentage of cells expressing the gene; color represents scaled expression of the gene. (**C**) Proportion of immune cell types in blood and bladder. Left: Box-and-whisker plots showing the distribution of immune cell type proportions for each patient, measured by Welch’s *t* test. Right: Stacked bar plots summarizing immune cell type proportions after downsampling to 200 cells per sample and measured by a χ^2^ test. (**D**) Proportion of immune cell types in the bladder tissue of BCG-naive, -responsive, and -unresponsive groups, measured by Welch’s *t* test. Left: Box-and-whisker plots showing the distribution of immune cell type proportions. Right: Stacked bar plots summarizing immune cell type proportions after downsampling to 200 cells per sample and measured by a χ^2^ test. Box-and-whisker plots depict median, IQR, and whiskers extending to 1.5× IQR in **C** and **D**. *P < 0.05, **P < 0.01, ***P < 0.001, ****P < 0.0001.

scRNA-seq data were generated for 84,616 cells using the BD Rhapsody single-cell system, with matched tissue and blood samples multiplexed together using sample tags. The BD system was selected to allow for the preservation of fragile neutrophil populations from the bladder (22). After quality control analyses, data were analyzed for 74,651 immune cells. We utilized the R package Seurat to integrate the data from 20 blood and 20 bladder biopsies across BCG naive, BCG responder, and BCG nonresponder groups using canonical correlation analysis. After integrating the samples, we projected the immune cells in 2 dimensions by UMAP and performed unsupervised clustering (Figure 2A). We identified immune cell populations using differentially expressed genes across these identified clusters (Figure 2B and Supplemental Figure 1A; supplemental material available online with this article; https://doi.org/10.1172/JCI200442DS1). To confirm our sequencing data quality, we plotted cell counts and the distribution of cell types for each patient in the tissue (Supplemental Figure 2A) and blood (Supplemental Figure 2B) biopsies. We next examined how immune cell composition varied between the peripheral blood and the tumor site (Figure 2, C and D, and Supplemental Figure 1B). A global comparison of all CD45^+^ cells revealed striking compartment-specific differences of immune cells between bladder tissue (*n* = 32,873) and blood (*n* = 41,778). We identified that blood samples predominantly comprised neutrophils (81% of cells), while T cells were the primary CD45^+^ cells isolated from the bladder (58% of cells) (Figure 2C). Underscoring the distinct immune landscape at the tumor site, the bladder tissues exhibited significantly higher frequencies of NK cells, CD4^+^ and CD8^+^ T cells, DCs, mast cells, and B cells compared with blood (Figure 2C). To understand how BCG influences the immune profile, we categorized samples based on BCG exposure status (pre-BCG [naive] and post-BCG [BCG responsive or unresponsive]) and compared the relative frequencies of conditions across immune cell types (Figure 2D). In the bladder, BCG exposure was linked to a notable increase in CD4^+^ T cells (Figure 2D), while the peripheral blood showed no significant change in overall immune composition (Supplemental Figure 1C). However, this increase in CD4^+^ T cells was mainly driven by BCG-responsive tumors, indicating a localized, treatment-related expansion of this subset in effective immune responses (Figure 2D).

### Opposing Th17 and Treg signatures define response to BCG.

**Figure 3:**
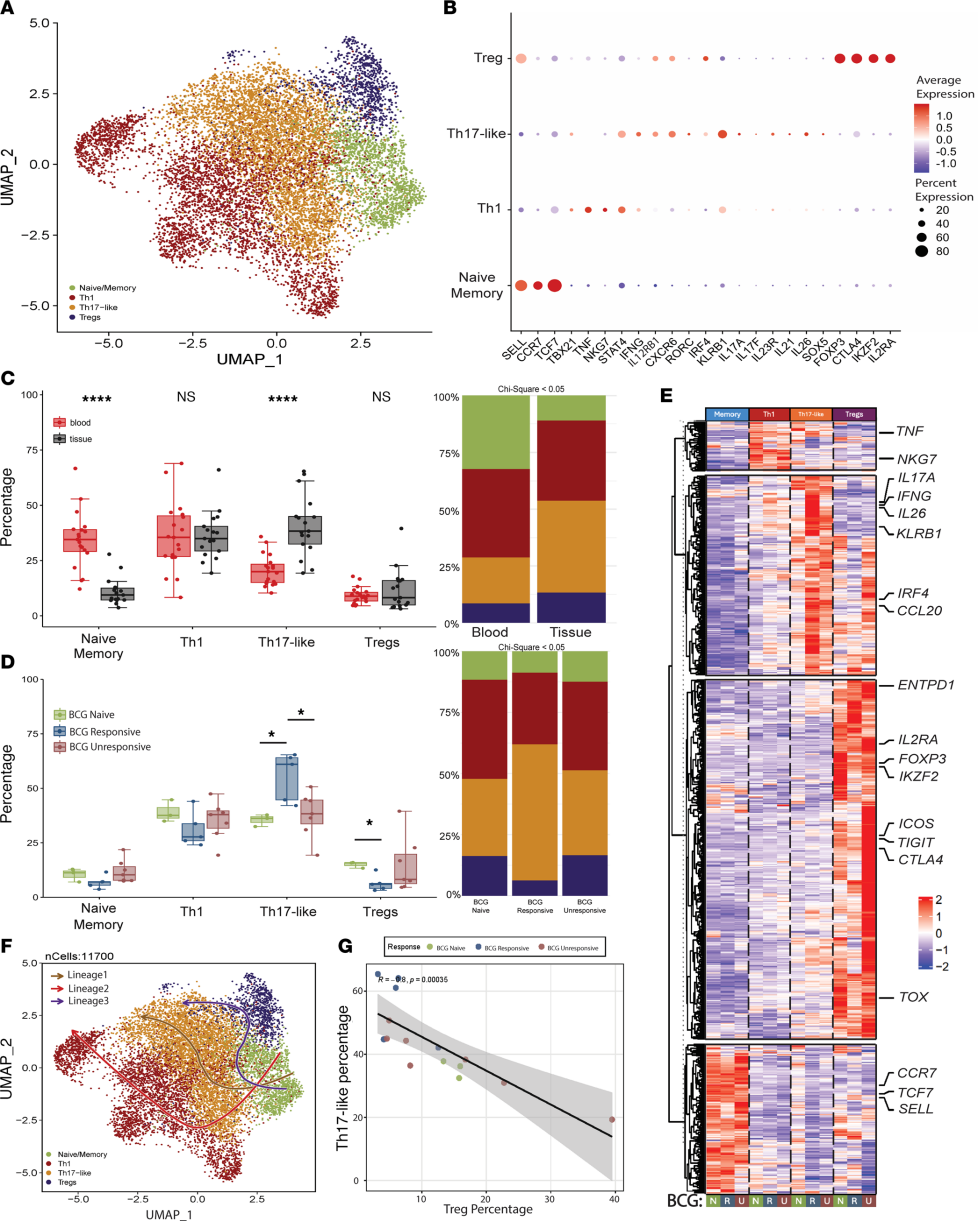
Opposing Th17 and Treg signatures define response to BCG. (**A**) UMAP plot of CD4^+^ T cells from the blood and bladder tissue of BCG-naive, -responsive, and -unresponsive groups. (**B**) Dot plot of representative genes expressed in each major cluster. Dot size represents percentage of cells expressing the gene; color represents scaled expression of the gene. (**C**) Proportion of CD4^+^ cell subsets in blood and bladder, measured by Welch’s *t* test. Left: Box-and-whisker plots showing the distribution of immune cell type proportions for each patient. Right: Stacked bar plots summarizing immune cell type proportions after downsampling to 100 cells per sample. (**D**) Proportion of CD4^+^ cell subsets in the bladder tissue of BCG-naive, -responsive, and -unresponsive groups, measured by Welch’s *t* test. Left: Box-and-whisker plots showing the distribution of CD4^+^ cell subset proportions. Right: Stacked bar plots summarizing CD4^+^ cell subset proportions after downsampling to 100 cells per sample. (**E**) Heatmap showing differentially expressed genes of CD4^+^ T cell subsets in BCG-naive (N), -responsive (R), and -unresponsive (U) groups. (**F**) Slingshot trajectory plot showing predicted cellular differentiation possibilities of CD4^+^ T cells. (**G**) Linear regression between a patient’s proportion of Th17-like and Treg cells. Each point represents 1 patient. Gray shaded region, 95% CI. Box-and-whisker plots depict median, IQR, and whiskers extending to 1.5× IQR in **C** and **D**. *P < 0.05, ****P < 0.0001.

**Figure 4:**
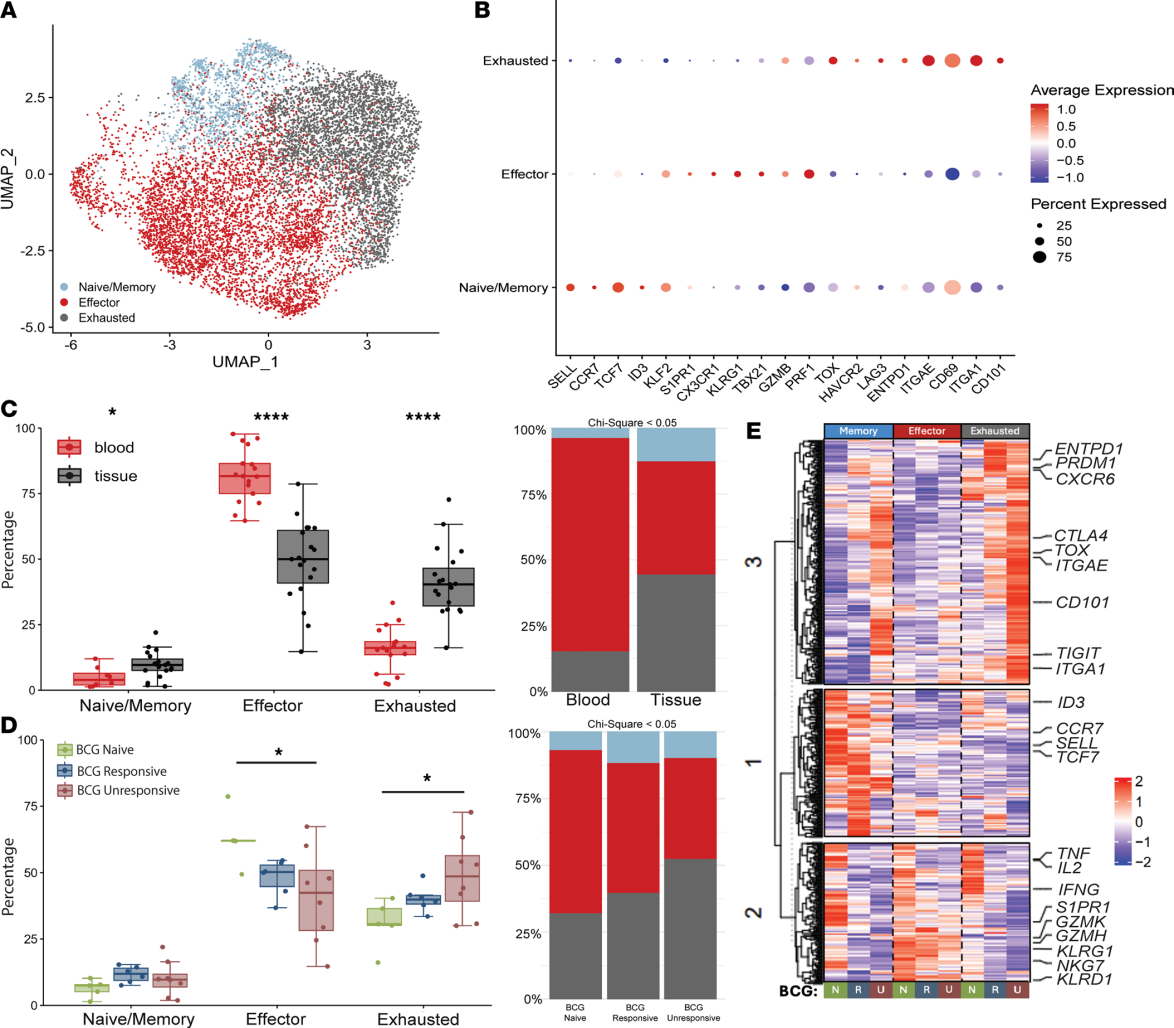
CD8^+^ T cells demonstrate increased exhaustion in BCG-unresponsive bladders. (**A**) UMAP plot of CD8^+^ T cells from the blood and bladder tissue from BCG-naive, -responsive, and -unresponsive groups. (**B**) Dot plot of representative genes expressed in each major cluster. Dot size represents percentage of cells expressing the gene; color represents scaled expression of the gene. (**C**) Proportion of CD8^+^ cell subsets in blood and bladder, measured by Welch’s *t* test. Left: Box-and-whisker plots showing the distribution of immune cell type proportions for each patient. Right: Stacked bar plots summarizing CD8^+^ subset proportions after downsampling to 200 cells per sample. (**D**) Proportion of CD8^+^ cell subsets in the bladder tissue of BCG-naive, -responsive, and -unresponsive groups, measured by Welch’s *t* test. Left: Box-and-whisker plots showing the distribution of CD8^+^ T cell subset proportions. Right: Stacked bar plots summarizing CD8^+^ cell subset proportions after downsampling to 200 cells per sample. (**E**) Heatmap showing differentially expressed genes of CD8^+^ T cell subsets categorized by BCG-naive, -responsive, and -unresponsive groups. Box-and-whisker plots depict median, IQR, and whiskers extending to 1.5× IQR in **C** and **D**. *P < 0.05, ****P < 0.0001.

Because they represent the greatest abundance of immune cells in the bladder, we first analyzed the lymphocyte pool including CD4^+^ (Figure 3), CD8^+^ (Figure 4), and NK cells (Supplemental Figure 3). We further examined the CD4^+^ T cell landscape before and after BCG administration since we identified a significant expansion of CD4^+^ T cells in the bladders of BCG-responsive patients. Overall, there were 11,757 CD4^+^ T cells across all patients and cellular compartments. To more accurately align the CD4^+^ T cells, we performed integration using SCTransform, followed by a UMAP projection and nearest-neighbor clustering (Figure 3A). To identify cell subtypes, we performed differential gene expression and identified previously described canonical CD4^+^ T cell types. The cluster with high expression of *TCF7*, *SELL*, *LEF1,* and *CCR7* was identified as “naive/memory cluster,” while the cluster with high expression of *FOXP3*, *IL-2RA*, and *IKZF2* was identified as “Tregs” (Figure 3B). Most other CD4^+^ T cells exhibited a Th1 phenotype, characterized by high levels of *STAT4*, *IL-12Rβ2*, and *IFN-γ*. However, some of these cells exhibited a more conventional Th1 profile, characterized by high expression of *TNF*, *TBX21*, and *NKG7*, while others displayed a unique expression of Th17-like markers, including *IL-17A*, *IL-17F*, *SOX5*, and *IL-26* (Figure 3B). To further investigate the differences between these CD4^+^ T cells, we applied a cosine similarity index, which performs pairwise comparisons of gene expression between cell types. We found that Tregs and naive/memory CD4^+^ T cells had little similarity to all other CD4^+^ cell subtypes, while Th1 and Th17-like Th1 cells had high pairwise cosine similarity scores, suggesting that these Th17-like cells are still part of the Th1 lineage rather than fully differentiated Th17 cells (Supplemental Figure 4A).

To analyze the immune profile of CD4^+^ T cells during BCa treatment with BCG, we compared the cell types between blood and bladder tissue (Supplemental Figure 4B). While the proportion of Th1 cells was consistent between treated and untreated bladder samples (~40%), Th17-like Th1 cells were markedly enriched in the bladder, emerging as the dominant CD4^+^ population (~45%) (Figure 3C). This shift was accompanied by a significant relative absence of naive/memory CD4^+^ T cells, reflecting a transition toward a more activated immune environment in the bladder (Figure 3C). We next determined how BCG exposure status impacted CD4^+^ T cell distributions. While BCG exposure had minimal impact on circulating immune cell populations (Supplemental Figure 4C), we found that BCG-responsive patients had increased Th17-like Th1 cells and decreased Tregs when compared with BCG-naive patients (Figure 3D). While Th1 changes have been described after BCG exposure, the Th17-like signature in BCG-responsive patients is consistent with the activation of a Th17 CD4^+^ T cell population after *Mtb* exposure (23).

To more narrowly explore how BCG exposure and response impact tissue-specific CD4^+^ T cells after BCG exposure, we investigated how gene expression was altered within these identified cell states. We identified the top differentially expressed genes and performed downsampling to ensure each patient was weighted equally. We then applied k-means hierarchical clustering to determine broad gene expression signatures. We identified 4 major gene signatures corresponding to each of the 4 major cell types identified in our UMAP clustering (Figure 3E). While many cell type–specific genes were expressed uniquely in each cluster, we also observed differences in gene expression between BCG exposures. Interestingly, we observed that the BCG-responsive group exhibited high gene expression of Th17 genes, such as *IL-17A* and *IL-26*, as well as higher expression of Th1 genes, including *IFNG*, within the Th17-like Th1 cell cluster. Together, this suggests that the Th17-like Th1 population is not only more frequent in BCG responders but also has a greater capacity to produce critical functional markers, indicating greater activation and function on a per-cell basis. In contrast, nonresponding BCG patients had higher expression of Treg signature genes, including *CTLA4*, *ICOS*, *FOXP3*, and *TOX*. Higher expression of these immunosuppressive surface markers, cytokines, and transcription factors suggests that BCG-unresponsive patients have Tregs with more potent immunosuppressive capacity. Th17 cells and Tregs have been previously shown to have mutual antagonism, competing for TGF-β during differentiation and subsequently establishing opposing inflammatory environments, a phenomenon well established in autoimmune diseases (24). This finding aligns with past work that identified a relationship between CD4^+^ T cells and BCa, whereby the percentage of IL-17–producing CD4^+^ T cells inversely correlates with the percentage of Treg cells (25). To explore this dynamic in our BCG BCa model, we performed trajectory analysis with the slingshot algorithm and identified 3 distinct lineages (Figure 3F). We observed that these lineages originated from the naive/memory CD4^+^ T cell population and differentiated into the Th1, Th1/17, and Treg populations. Overall, our model suggests an early branch point prior to terminal fate commitments. Consistent with the mutual antagonism model of differentiation, we observed a negative correlation between Th1/17 and Treg populations across samples (Figure 3G). Together, our results suggest that this Th17/Treg relationship is critical for generating effective immune responses to mycobacterial infections such as BCG and *Mtb*.

### CD8^+^ T cells demonstrate increased exhaustion in BCG-unresponsive bladders.

While there were no significant differences in the overall proportion of CD8^+^ T cells between responders and nonresponders to BCG (Figure 2, C and D), we sought to identify differences in CD8^+^ T cell subsets using single-cell profiling. We isolated the CD8^+^ T cell population, reintegrated the cells, and applied unbiased UMAP clustering, where we identified 3 distinct clusters through shared nearest-neighbor optimization (Figure 4A). These CD8^+^ subtypes had varied cell expressions that reflected previously described cell types including naive/memory T cells (*TCF7*, *KLF2*, *SELL*, and *CCR7*), effector T cells (*KLRG1*, *PRF1*, and *CXC3R1*), and exhausted T cells (*TOX*, *ENTPD1*, and *LAG3*) (Figure 4B and Supplemental Figure 5A) (26, 27). To explore how bladder tissue affects the CD8^+^ T cell populations, we compared the shifts in population between the blood and bladder. We observed a higher number of effector CD8^+^ T cells in the blood and an enrichment of exhausted CD8^+^ T cells in the bladder (Figure 4C and Supplemental Figure 5B). This tissue-specific shift in CD8^+^ T cell profile suggests BCa promotes localized T cell exhaustion.

To determine if BCG-responsive and -unresponsive patients had differences within CD8^+^ T cells, we compared the frequency of CD8^+^ cell subtypes. While there was no difference in CD8^+^ cell types in the blood (Supplemental Figure 5C), we identified significantly lower effector CD8^+^ T cells in BCG-unresponsive bladders and significantly increased exhausted CD8^+^ T cells in BCG-unresponsive bladders (Figure 4D). To more specifically explore how BCG exposure impacts CD8^+^ T cell differentiation, we analyzed the differential expression of individual genes within each cell across both CD8^+^ clusters and BCG exposures, identifying 1,064 genes. To control differences in patient cell count, each patient sample was randomly downsampled to 200 cells to ensure equal weighting between patient replicates. We then applied k-means hierarchical clustering so that we could identify broad gene expression signatures (Figure 4E). We found that gene expression could be categorized into 3 main signatures, namely, Tpro, Teff, and Texh. While these signatures corresponded to CD8^+^ T cell clusters, the signatures were not equally shared across BCG exposures within those clusters. In the naive/memory cluster, the Tpro signature was more highly expressed in the BCG naive and BCG responders, while the BCG nonresponders indicated a lower level of expression. Notably, recent investigations of CD8^+^ T cells have highlighted the significance of these stem-like signatures in sustaining robust CD8^+^ T cell responses against tumors (28, 29). The Teff gene cluster exhibited a shared signature of effector-related genes, including *GZMK*, *GZMH*, *KLRG1*, *S1PR1*, and *NKG7,* across the BCG exposures. However, a second effector signature comprising cytokines such as *TNF*, *IL-2*, and *IFNG* was specific to the BCG-naive condition, with a significant reduction in patients exposed to BCG. Finally, we identified a third signature made up of genes related to exhaustion. Overall, we found that some genes were shared across BCG exposures in the exhausted cluster, including *ENTPD1*, *CXCR6*, and *PRDM1*. However, the BCG-nonresponding patients showed significantly higher expression of additional exhaustion markers, including *CTLA4*, *TOX*, and *TIGIT*, as well as tissue residence–related markers, including *ITGAE*, *CD101*, and *ITGA1*. Notably, we found that this exhausted CD8^+^ T cell signature was also higher in the naive/memory cluster, indicating that these precursor cells may already be undergoing exhaustion. Interestingly, the anti–PD-1 therapy function is known to operate by inhibiting PD-1 on these progenitor cells (30). These results suggest that corresponding immunotherapies may be beneficial for BCG-nonresponding patients who exhibit high levels of CD8^+^ T cell exhaustion. Overall, this analysis reveals that BCG-nonresponding patients exhibit altered CD8^+^ T cell differentiation and cellular signatures characteristic of T cell exhaustion.

To examine immune cell signatures more closely in BCG BCa, we sampled 11 separate bladder biopsies on patients not included in the scRNA-seq study. We designed a flow cytometry panel to examine NK and CD8^+^ T cell effector signatures and found that BCG responders had more TNF-α^+^CD8^+^ T cells, whereas nonresponders had an increased percentage of TNF-α/IFN-γ double-negative CD8^+^ T cells (Supplemental Figure 5D). These findings complement our single-cell findings by suggesting that CD8^+^ T cells exhibit increased cytotoxic function in BCG responders.

### Macrophage polarization and T cell interactions.

**Figure 5:**
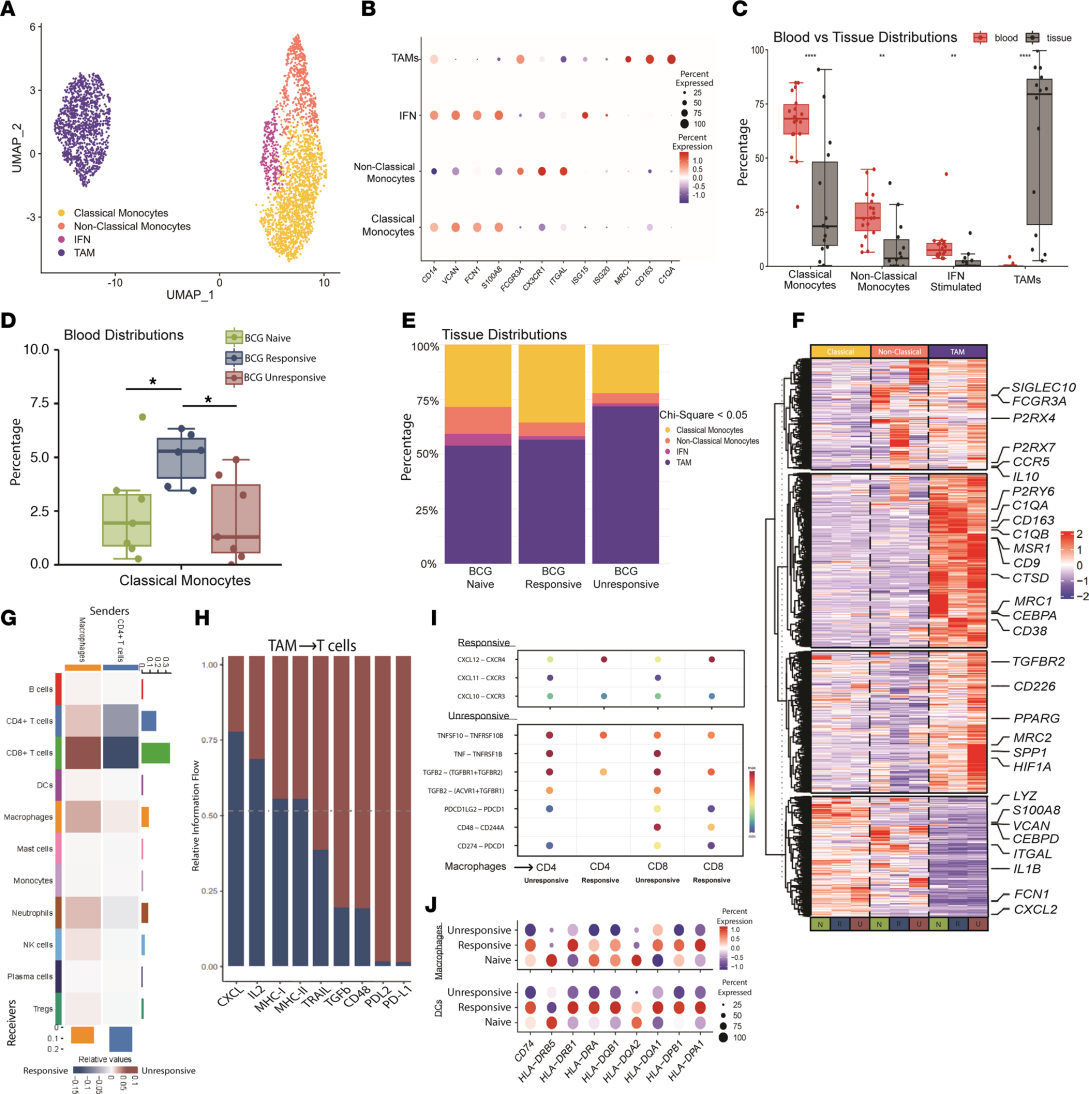
Macrophage polarization and T cell interactions. (**A**) UMAP plot of monocyte and macrophage cells from the blood and bladder tissue of BCG-naive, -responsive, and -unresponsive groups. (**B**) Dot plot of representative genes expressed in each major cluster. Dot size represents percentage of cells expressing the gene; color represents scaled expression of the gene. (**C**) Proportion of monocyte and macrophage subsets between the blood and bladder measured, by Welch’s *t* test. Each dot represents 1 patient. (**D**) Proportion of monocytes and macrophages in the blood of BCG-naive and -exposed (responsive and unresponsive) groups measured, by Welch’s *t* test. (**E**) Stacked bar plots summarizing monocyte and macrophage cell subset proportions after downsampling to 50 cells per sample in the bladder tissue. Significance measured by χ^2^ test. (**F**) Heatmap showing differentially expressed genes of monocyte and macrophage subsets categorized by BCG-naive, -responsive, and -unresponsive groups. (**G**) Heatmap of CellChat’s cell–cell interaction scores between BCG-responsive and-unresponsive cells. (**H**) Differences in TAMs to T cell interaction scores between BCG-responsive and -unresponsive cells. (**I**) Dot plot of macrophage ligands/cytokines to T cell receptors that are increased (lower) and decreased (upper) in BCG nonresponders. Color represents communication probability of a ligand/receptor signaling pathway. (**J**) Dot plot of MHCII genes in APCs. Dot size represents percentage of cells expressing the gene; color represents scaled expression of the gene. Box-and-whisker plots depict median, IQR, and whiskers extending to 1.5× IQR in **C** and **D**. *P < 0.05, **P < 0.01, ****P < 0.0001.

We further evaluated the myeloid populations, including macrophages (Figure 5) and neutrophils (Supplemental Figure 6). *Mtb* primarily infects alveolar macrophages in the lung, and numerous studies have implicated macrophage-mediated signaling as a central mechanism of tuberculosis-driven immune regulation (31, 32). Given the parallels between *Mtb* infection and intravesical BCG therapy, we sought to investigate how BCG influences the myeloid compartment within the bladder. To investigate this, we reintegrated all monocyte and macrophage cells, applied UMAP dimensionality reduction, and performed nearest-neighbor clustering, which revealed 4 major clusters (Figure 5A). Myeloid cell populations were primarily composed of classical monocytes (*CD14* and *VCAN*) and nonclassical monocytes (*FCGR3A*, *CX3CR1*, and *ITGAL*) (Figure 5B). Some cells shared the expression signature of classical monocytes but also had a strong interferon-stimulated signature (*ISG15* and *ISG20*) (Figure 5B). Finally, a cluster of macrophages with a primarily M2-like phenotype was identified (*CD163* and *MRC1*), which we termed tumor-associated macrophages (TAMs) (Figure 5B). Having defined these distinct myeloid subsets, we sought to understand how their distribution differed between systemic circulation and the bladder microenvironment (Supplemental Figure 7A). Reflecting the capacity of tissue environments to drive monocyte-to-macrophage differentiation, we found that bladder tissue exhibited a reduction in circulating monocyte populations and a notable expansion of macrophages (Figure 5C). To explore how BCG influences the myeloid compartment, we compared myeloid populations between BCG-naive and BCG-exposed patients. Interestingly, classical monocytes, which are primarily involved in inflammatory responses, were increased in the blood of BCG-responsive patients (Figure 5D and Supplemental Figure 7B) (33, 34). Overall, this shift suggests that BCG responders induce a systemic bias toward an inflammatory monocyte phenotype, which in turn modulates the tumor microenvironment upon tissue infiltration and macrophage differentiation.

To explore how BCG impacts macrophage differentiation in the bladder, we examined overall macrophage populations. We found that the majority (1,071 cells) of the total macrophages (1,284 cells) were present in the BCG-unresponsive bladders, with a limited number of macrophages (213 cells, 17%) found in the BCG-responsive bladders. While we saw a trend toward greater TAMs across BCG conditions within the bladder tissue, there was a high degree of heterogeneity across patients resulting in no significant differences across groups (Supplemental Figure 7C). However, when we performed downsampling and quantified cellular differences, we noticed that BCG-unresponsive patients had the highest proportion of TAMs (Figure 5E). TAMs have been described as having both proinflammatory and immunosuppressive roles. Although we did not observe discrete subsets corresponding to classically defined M1 and M2 macrophages, we hypothesized that the overall macrophage compartment could still exhibit phenotypic polarization between BCG responders and nonresponders. To test this, we downsampled to control for interpatient differences in cell numbers and performed differential gene expression analysis between cells from BCG-naive, BCG-responsive, and BCG-unresponsive patients (Figure 5F). Notably, TAMs from BCG nonresponders had elevated expression of a distinct cluster of genes, such as *SPP1*, which has been implicated in regulation of T cell exhaustion, as well as *PPARG*, *TGFBR2*, and *MRC2*, which are associated with regulatory or immunosuppressive functions (35, 36).

To better understand whether TAMs orchestrate immune responses to BCG, we utilized the CellChat algorithm to analyze ligand–receptor interactions across various immune cell types. By weighing the total outgoing signaling from each population and comparing BCG responders to nonresponders, we observed notable shifts in cellular crosstalk (Figure 5G). While overall cell–cell interaction strength was largely comparable between groups, we identified striking differences in signaling directed toward CD8^+^ T cells. In BCG-unresponsive patients, CD8^+^ T cells received predominant input from TAMs, whereas in BCG-responsive patients, CD4^+^ T cells were the main signaling source (Figure 5G). This pattern aligns with recent evidence that CD4^+^ T cell help is essential for sustaining CD8^+^ T cell function and preventing exhaustion, while TAMs are implicated in regulation of CD8^+^ T cell dysfunction (26, 37, 38).

To dissect the pathways mediating these interactions, we compared CellChat signaling profiles between groups. PD-L1 and PD-L2 signaling were the most enriched inhibitory pathways in BCG-unresponsive patients, accompanied by elevated TIGIT, CD244A, and CD266 signaling, as well as immunosuppressive cytokines including TNFSF10 (TRAIL) and TGF-β (Figure 5, H and I). In contrast, BCG-responsive patients exhibited increased signaling through proinflammatory and recruitment pathways, including CXCL9, CXCL10, CXCL11, CXCL12, and CXCL16, along with enhanced IL-2 and MHCII pathways (Figure 5, H and I). Interestingly, BCG is known to suppress MHC expression (39), and MHCII expression was not exclusive to BCG-responding TAMs but was more highly expressed across all BCG-responding antigen-presenting cells (Figure 5J and Supplemental Figure 8A). To validate this finding, we analyzed our previously published bulk RNA-seq dataset from 103 BCG-treated stage I NMIBC tumors (40). Higher expression of antigen presentation genes such as *CD74* and *HLA-DOA* was associated with significantly improved survival, supporting the importance of high MHC signaling for effective responses (Supplemental Figure 8B).

To further investigate how CD4^+^ T cells may support CD8^+^ T cell function in BCG responders, we specifically examined signaling between these 2 populations. While no signaling pathways were strongly enriched in unresponsive patients, we identified a significant enrichment of the CCL5 (RANTES)/CCR5 axis in BCG responders (Supplemental Figure 8C). Although IL-21 signaling is widely recognized as a central mediator of CD4^+^ T cells to CD8^+^ T cells, recent studies have shown that RANTES also contributes to the differentiation of CD8^+^ T cells into the effector lineage (41).

To validate some of our predicted interactions, we performed multiplex immunofluorescence using 30 markers on 3 BCG-responsive and 3 BCG-unresponsive tissue samples. We utilized QuPath to generate a single measurement classifier to determine set thresholds of signal positivity to characterize immune cell types such as DCs, macrophages, and CD4^+^ and CD8^+^ T cells. Grossly, BCG responders exhibited greater immune cell infiltration, often forming lymphoid aggregates (Supplemental Figure 9, A–C). We then used the R package Spatial Image Analysis of Tissues to determine localization differences between different cell types. We identified that CD4^+^ T cells were closer to TAMs in nonresponders than in responders. To control potential inherent localization differences between tissues, we also compared CD4^+^ T cells to DCs and found no differences in cellular distance (Supplemental Figure 9, D and E). Together, this supports the CellChat findings of a TAM-driven immunosuppressive signaling network.

### Machine learning model to infer important cellular and gene characteristics of BCG responders.

**Figure 6:**
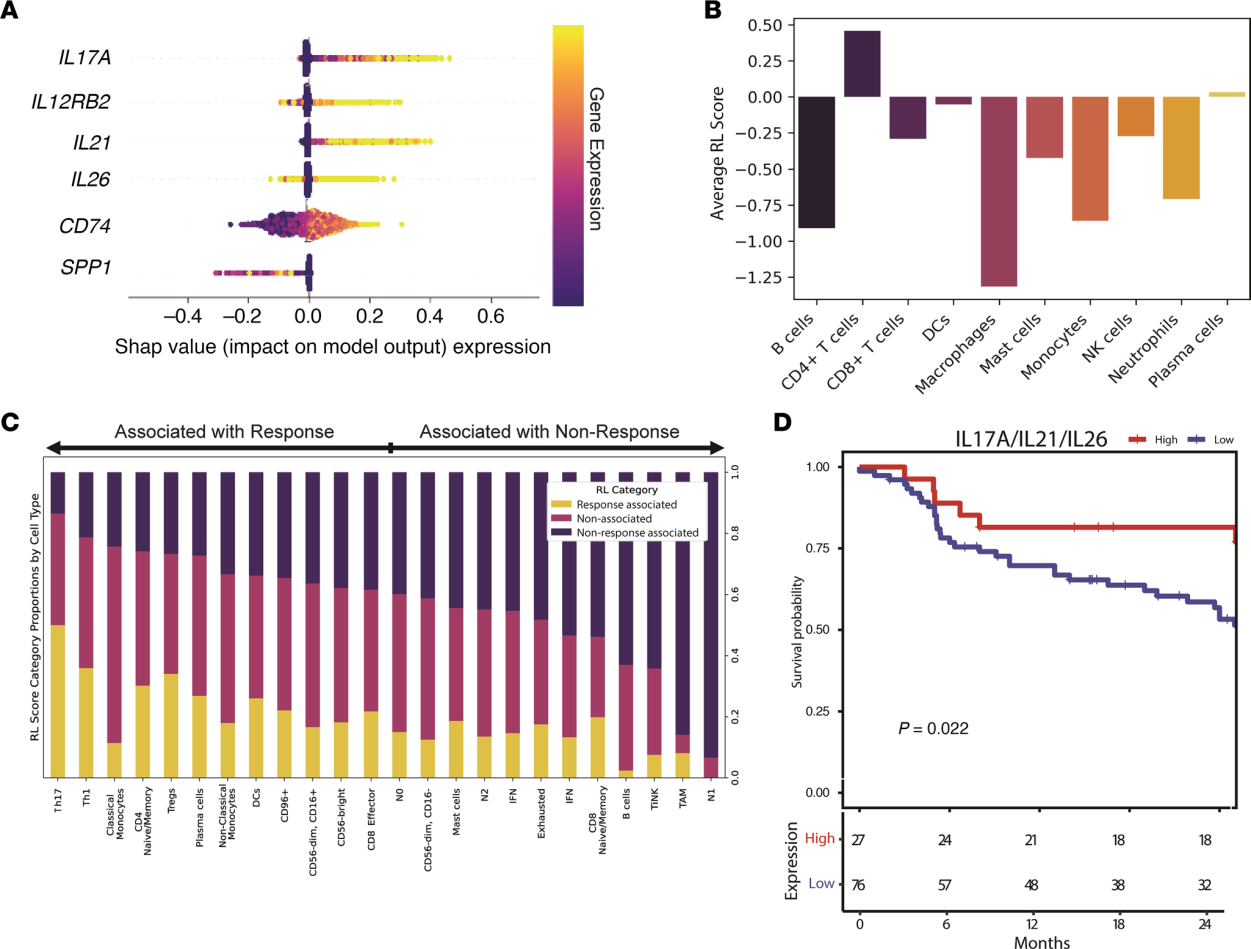
Machine learning model to infer important cellular and gene characteristics of BCG responders. (**A**) Representative Boruta-selected genes. Positive SHAP values indicate that the gene is predicative of BCG responders. (**B**) Reinforcement learning (RL) score averaged for each major cell type. Positive RL scores indicate that the cell type is predictive of BCG responders. (**C**) Proportion of a cell subtype’s RL score categorized by associated with response (>0.5) or nonresponse (<0.5) or nonassociated (–0.5, 0.5). (**D**) Kaplan-Meier curve of recurrence-free survival for patients expressing high and low expression of IL-21, IL-17A, and IL-26 defined by a log-rank *P* value.

Having constructed a single-cell atlas of the immune landscape in BCa, we next aimed to quantify the relative contribution of each immune cell type in predicting BCG responsiveness. We applied the Precise (Predictive Response Analysis from Single-Cell Expression) machine learning framework, which leverages the XGBoost algorithm in a leave-one-out cross-validation setup (42). Feature importance was refined using Boruta selection, ultimately identifying 57 highly informative genes (Supplemental Figure 10A). To further dissect the impact of these genes on model performance, we calculated SHAP (Shapley additive explanations) values, which quantify each gene’s contribution to predicting BCG response (Supplemental Figure 10B). Notably, CD4^+^ T cell–derived cytokines such as IL-17A, IL-21, and IL-26, along with the receptor IL-12RB2, were among the strongest positive model-informative features of response (Figure 6A). These findings align with our earlier observations, which implicated Th17-like Th1 cells in effective immune responses. Moreover, expression of *CD74*, a key component of antigen presentation by APCs, also positively influenced response classification (Figure 6A and Supplemental Figure 10B). In contrast, genes enriched in suppressive myeloid populations, such as *SPP1* in TAMs, contributed to classification of BCG nonresponsiveness (Figure 6A). Together, this integrative analysis highlights both molecular signatures that distinguish responders from nonresponders and provides a framework for future biomarker development.

To determine which immune cell types are most critical for therapeutic outcomes, we applied Precise’s reinforcement learning framework using the Boruta-selected gene list. In this approach, initial cell labels were assigned as +1 for cells from BCG responders and −1 for those from nonresponders. These labels were then iteratively updated based on the model’s accuracy in classifying each cell to outcome, allowing us to quantify the classification contribution of each cell type. Remarkably, CD4^+^ T cells emerged as the only population with a net positive classification value for BCG response, reinforcing their unique role in driving effective antitumor immunity (Figure 6B). In contrast, while multiple cell types contributed to classifying nonresponse, macrophages stood out as the strongest indicators of treatment failure (Figure 6B). This dichotomy suggests that an effective BCG response hinges on the presence of immunostimulatory CD4^+^ T cell subsets, whereas an immunosuppressive myeloid environment dominated by macrophages may underlie therapeutic resistance.

To better understand which immune populations are associated with BCG treatment outcomes, we evaluated each cell subtype based on categorizations of the reinforcement learning scores. We first assigned a classification to each cell individually. Cells scoring less than –0.5 were considered “associated with nonresponse” and those with a score greater than +0.5 as “associated with response.” Cells with scores near zero, indicating no predictive power, were termed “nonassociated.” Ranking cell types by these categorizations revealed patterns consistent with known biology (Figure 6C). For instance, exhausted CD8^+^ T cells were more strongly linked to BCG nonresponse, whereas effector CD8^+^ T cells were associated with response (Figure 6C). Similarly, proinflammatory classical monocytes and cytotoxic CD16^+^ NK cells had higher response association scores than their immunosuppressive counterparts, such as tumor-infiltrating NK cells and TAMs (Figure 6C). Notably, Th17-like Th1 cells emerged as the most important subset for classifying BCG responsiveness (Figure 6C). Together, these results underscore how the overall immune cell composition shapes the trajectory of BCG treatment response.

Given that Th17-like Th1 CD4^+^ T cells were the most enriched population in BCG responders and that IL-17A, IL-21, and IL-26 emerged as top predictive features in our machine learning analysis, we sought to validate this observation in an independent dataset. Using our previously published cohort of 103 BCG-treated patients, we stratified individuals based on their cytokine expression. Strikingly, patients with high expression demonstrated significantly improved recurrence-free survival over 24 months, with 18 out of 27 (66%) remaining recurrence free compared with only 32 out of 76 (42%) in the low expression group (Figure 6D). These results suggest that a robust Th17-like Th1 response may play a pivotal and previously underappreciated role in orchestrating effective antitumor immunity in the context of BCG therapy.

## Discussion

In this study, we define the immune landscape of the bladder during intravesical BCG therapy using single-cell transcriptomic profiling of bladder-infiltrating and circulating immune cells from patients with high-risk NMIBC. Recent investigations in BCG BCa have begun single-cell cellular profiling and have identified potential cell–cell interactions regulating BCG efficacy (43). By integrating cell state analysis, cell–cell communication, and machine learning, our work identifies distinct immune programs that segregate BCG-responsive from -nonresponsive patients. Our data highlight a Th17-like Th1 CD4^+^ T cell subset, robust antigen presentation, and reduced immunosuppressive signaling as hallmarks of BCG responsiveness. In contrast, BCG unresponsiveness is characterized by Treg dominance, exhausted CD8^+^ T cells, and macrophage-driven immunosuppression.

While the exact immune-driven mechanism of BCG in BCa is not fully understood, it likely follows a similar path to the conventional mechanism underlying control of mycobacterial infections such as *Mtb*. BCG is an attenuated form of *Mtb* due to deletion of the RD1 encoding the ESX-1 type VII secretion system (12, 13). Here, it is understood that mycobacteria are taken up by macrophages, where antigen presentation through MHC molecules and secretion of IL-12 promote differentiation of Th1 CD4^+^ T cells. These cells produce IFN-γ and TNF, which promote the formation of granulomas, organized structures of chronically activated macrophages encircled by lymphocytes that continuously preserve granuloma integrity. In *Mtb*, such immune complexes serve to contain mycobacteria in a latent state, and in BCa, they likely function as a localized hub of sustained immune activation. Supporting this, histological studies of BCG-treated tissue have found that granuloma formation correlates with improved recurrence-free survival (44, 45).

BCG treatment is known to increase circulating monocytes and drive macrophage infiltration into the bladder wall, and circulating monocytes are elevated in the urine (46). Clinically, a higher density of CD68^+^ macrophages in tumors following intravesical BCG immunotherapy correlates with improved recurrence-free survival in NMIBC patients (46). Paradoxically, however, elevated numbers of TAMs prior to treatment are linked to poor outcomes, suggesting that macrophage origin and polarization state critically shape therapeutic efficacy (46). As evidenced by *Mtb* infections, granuloma formation is primarily sustained by M1-polarized activated macrophages in latent disease, whereas suppressive M2 polarization correlates with an uncontrolled active disease state (47). By analogy, resident TAMs in the bladder tumor microenvironment are thought to be primarily in an immunosuppressive state, whereas newly recruited monocytes differentiate into more activated macrophages that promote tumor control (33, 34). In line with these findings, recent investigations suggest that the protective effects of BCG are mediated, at least in part, through immune reprogramming within the bone marrow, where hematopoietic cells acquire memory-like properties that subsequently contribute to antitumor immunity (48, 49). Our data support these models, as we observed that BCG responders not only had more activated gene signatures in TAMs, but also had significantly higher frequencies of classical monocytes in their blood. These classical monocytes are known to differentiate into more functional macrophages, whereas nonclassical monocytes give rise to more immunosuppressive macrophages (33, 34). This increase in classical monocytes, together with the reduction in macrophage immunosuppression signature in BCG responders, suggests that recruitment and differentiation of fresh monocytes into inflammatory macrophages is critical for antitumor activity. Consistent with this notion, studies in B16 melanoma have shown that intravenous BCG induces monocyte populations that differentiate into inflammatory macrophages essential for tumor control and that transfer of BCG-conditioned bone marrow is sufficient to protect against tumor growth (50). Together, these findings highlight monocyte-derived macrophages as key mediators of effective BCG immunotherapy outcomes.

Macrophages then orchestrate the adaptive immune responses by presenting antigens and shaping the differentiation of CD4^+^ and CD8^+^ T cells through cell–cell interactions. We found that BCG-nonresponding TAMs had increased immune checkpoint inhibitor interactions with T cells through PDL1, PDL2, TGFb, CD48, and TRAIL. Since such markers have been found to influence the differentiation of T cells, these interactions suggest that macrophages contribute to the exhausted lymphocyte profile of BCG-unresponsive patients, which is characterized by a higher proportion of exhausted CD8^+^ T cells and Tregs. Rather than expression by the bladder epithelium, this suggests that bladder resident macrophages could be a source of exhaustion after BCG.

By comparison, the macrophages in BCG responders had a less suppressive phenotype, with markedly higher expression of MHCII genes. Since *Mtb* and BCG have a primarily phagosomal life cycle, adaptive immune identification is chiefly mediated through presentation of peptide:MHCII (51). This emphasizes the likely importance of the CD4^+^ T cell compartment in BCG response and why HIV infection is such a strong risk factor for tuberculosis (52). A key observation we found was the emergence of Th17-like Th1 CD4^+^ T cells in BCG-responsive patients that underwent potent expansion. These cells expressed both canonical Th1 molecules (e.g., IFNG, STAT4, and TBX21) and Th17-associated cytokines (IL-17A, IL-17F, IL-26, and IL-21). Due to the low number of macrophages, we could not comprehensively examine lowly expressed cytokines that likely play a role in this differentiation process, such as IL-12 or IL-6. While we could not identify a definitive interaction with macrophages that was implicated in this differentiation, prior studies in other mycobacterial models have found that M1-like macrophages induce Th1 and Th17 cell responses (53). Together, this suggests that proinflammatory macrophages in BCG responders could be playing a key role in this differentiation.

In addition, we found that these Th17-like Th1 cells were not only more abundant in responders, but also were the gene signature enriched in our machine learning model, suggesting they represent a qualitatively superior population in mounting local antitumor immunity. In particular, the cytokines IL-21, IL-26, and IL-17A were identified as the most critical for classification of BCG responsiveness in our machine learning model, which was subsequently validated in a separate cohort. These genes have several complex and conflicting roles; for example, IL-17A has been correlated with higher-grade tumors and is known to promote angiogenesis (54). By contrast, early expression of IL-17A is critical for recruitment of neutrophils (55). Alternatively, Th17 cells are known to compete with Tregs for TGF-β, so the generation of Th17 cells could simply be a way to prevent the development of immunosuppressive Tregs (24). While this Th17-like Th1 cell’s role in BCG response is not completely understood, studies in *Mycobacterium leprae* have found that Th17 gene signatures are correlated with reduced *M*. *leprae* burden (56). Finally, we found that these Th17-like Th1 cells produced higher levels of IL-21 and CCL5 (also known as RANTES). These cytokines exemplify classic CD4^+^ T cell “help,” whereby CD4^+^ T cells provide supportive signals, through cytokines and costimulatory interactions, that enhance CD8^+^ T cell activation, proliferation, and effector differentiation (26, 41). Our cell–cell interaction analysis, together with prior literature, suggests that IL-21 and RANTES from Th17-like Th1 cells contribute to the priming and functional maturation of CD8^+^ T cells into cytotoxic effector cells.

Finally, we acknowledge limitations to these findings. While this investigation is one of the largest single-cell analyses of BCG-treated bladders, an increased number of patients would potentially refine subset differences in cell types that had a limited number of cells and remove any batch effects identified in our machine learning model. Moreover, our cell–cell interactions were inferenced based on predicted interactions, and future investigations of multidimensional omics could validate the 3D nature of immune cell interactions. In addition, while immune profiling was performed on nontumor bladder tissue, we acknowledge that the broader bladder microenvironment may still be influenced by the presence of tumors elsewhere in the organ. Although our sampling strategy was specifically designed to reduce direct tumor-driven differences in immune infiltrates, we recognize that this represents an inherent limitation of human posttreatment sampling. Finally, biopsies were obtained prospectively and were not repeated evaluations of the same patients before and after BCG.

Our work identifies the heterogeneity and immune landscape associated with responses to BCG in NMIBC. We report that critical differences in T cell and myeloid cell populations are associated with clinical resistance to BCG and identify critical population changes and cellular interactions of interest for future investigation. Our results show that there are parallel signaling pathways between persistent *Mtb* infection of the lung and resistance to BCG therapy. Notably, preclinical and clinical studies, including PD-1 blockade trials in humans, have shown that enhanced T cell responses can increase susceptibility to TB (57–60), and multiple vaccine platforms designed to boost *Mtb*-specific T cell responses have consistently failed to confer protection against TB, despite generating robust antigen-specific responses (61, 62). Thus, while the same immune pathways may be deleterious in TB, they are well suited to promote tumor control, underscoring the context-dependent consequences of immune mechanisms. Future studies that leverage our identified population changes and cellular interactions may be applied to overcome BCG resistance and treatment response by risk stratification and combinatorial therapies.

## Methods

Additional details may be found in Supplemental Methods.

### Sex as a biological variable.

Male and female participants were included in the study; however, patients were prospectively enrolled for sample evaluation.

### Sample collection and processing.

Peripheral blood and tissue were collected from the patients at the time of surgery, immediately prior to standard-of-care BCa resection, and processed for scRNA-seq. It is our standard treatment paradigm to evaluate patients with a history of carcinoma in situ by blue light cystoscopy and bladder biopsy in the operating room after adequate BCG (FDA definition, 9 doses, including induction BCG [6 doses] and maintenance [3 doses]). Sample size was not determined prior to the prospective collection of samples, and response to BCG was determined by the pathology report after surgery. Each biopsy sample was < 1 cm in size. Enrolled participants had not been previously treated with BCG. BCG was initiated 4 weeks after the last diagnostic biopsy, and post-BCG biopsies were performed 6 weeks after the last BCG. All biopsies were of adjacent, normal-appearing bladder tissue.

### Statistics.

Statistical comparisons for sequencing data were performed in R. Comparisons were performed with the stat_compare_means function using a 2-sided *t* test with unequal variances with post hoc correction through the Benjamini-Hochberg procedure. Comparisons of cellular distributions in stacked bar plots were performed through a χ^2^ test. Linear regressions were performed using the lm function. A *P* value less than 0.05 was considered significant. Samples were occasionally excluded due to low cell counts from sequencing data. For all box-and-whisker plots where percentages were calculated in the tissue compartment, a minimum cell threshold was set at 100 cells per patient, and this cell threshold was reduced by increments of 25 cells until each BCG condition had at least 3 samples.

### Study approval.

This study was approved by the IRB of Northwestern University (STU00219216). Written informed consent was received prior to participation.

### Data availability.

scRNA-seq data are available in the Gene Expression Omnibus database with accession number GSE322495. The original R and Python codes used to generate most figure panels are publicly available on GitHub: https://github.com/ryanjbrown21/scBCG_immune_profiling (commit ID 14bf804fdea98136250e2f861264fe7c6f203b41). Any additional code may be requested from the corresponding author. Data used to perform calculations in the figures are provided in the Supporting Data Values file.

## Author contributions

Conceptualization: MTK and JJM. Methodology: RJB, MTK, JRP, WC, BC, and JJM. Software: RJB and MTK. Validation: RJB, MTK, WC, BC, and JRP. Formal analysis: JJM, RJB, AJH, HN, MTK, JRP, and BC. Investigation: all authors. Imaging: AH. Data curation: all authors. Writing (original draft): RJB, MTK, JJM, and JRP. Writing (review and editing): all authors. Visualization: RJB, MTK, JJM, and JRP. Supervision: JJM, JRP, BC, and WC. Funding acquisition: JJM.

## Conflict of interest

JJM participated in a Data and Safety Monitoring Board for Medscape and advisory boards or consulting for Merck, AstraZeneca, Janssen, BMS, UroGen, Prokarium, Imvax, Pfizer, Seagen/Astellas, Ferring, CG Oncology, Immunity Bio, Protara, and Photocure.

## Funding support

This work is the result of NIH funding, in whole or in part, and is subject to the NIH Public Access Policy. Through acceptance of this federal funding, the NIH has been given a right to make the work publicly available in PubMed Central.

- Veterans Health Administration grants BX005599 and BX003692 to JJM.
- Department of Defense Impact Award HT94252410507 to JJM.
- National Cancer Institute grant R01CA298333 to JJM.
- Robert H. Lurie Comprehensive Cancer Center H Foundation Core Facility Pilot Project Award to MTK.

## Supplementary Material

Supplemental data

ICMJE disclosure forms

Supplemental table 1

Supplemental table 2

Supporting data values

## Version 1 05/04/2026

In-Press Preview

## Version 2 06/15/2026

Electronic publication

## References

[1] Dobruch J, Oszczudłowski M (2021). Bladder cancer: current challenges and future directions. Medicina (Kaunas).

[2] Saginala K (2020). Epidemiology of bladder cancer. Med Sci (Basel).

[3] Gaylis FD (2024). Adherence to first-line intravesical bacillus calmette-guérin therapy in the context of guideline recommendations for US patients with high-risk non-muscle invasive bladder cancer. J Health Econ Outcomes Res.

[4] Sievert KD (2009). Economic aspects of bladder cancer: what are the benefits and costs?. World J Urol.

[5] Flaig TW (2022). NCCN guidelines insights: bladder cancer, Version 2.2022. J Natl Compr Canc Netw.

[6] Ourfali S (2021). Recurrence rate and cost consequence of the shortage of Bacillus Calmette-Guérin connaught strain for bladder cancer patients. Eur Urol Focus.

[7] Kodera A (2023). The management of Bacillus Calmette-Guérin (BCG) failure in high-risk non-muscle invasive bladder cancer: a review article. Cureus.

[8] Zlotta AR (2013). The management of BCG failure in non-muscle-invasive bladder cancer: an update. Can Urol Assoc J.

[9] Chehroudi AC, Black PC (2020). Emerging intravesical therapies for the management of bacillus Calmette-Guérin (BCG)-unresponsive non-muscle-invasive bladder cancer: charting a path forward. Can Urol Assoc J.

[10] Filon M, Schmidt B (2025). New treatment options for non–muscle-invasive bladder cancer. Am Soc Clin Oncol Educ Book.

[11] Harvey M (2022). Critical shortage in BCG immunotherapy: How did we get here and where will it take us?. Urol Oncol.

[12] Brosch R (2002). A new evolutionary scenario for the Mycobacterium tuberculosis complex. Proc Natl Acad Sci U S A.

[13] Hsu T (2003). The primary mechanism of attenuation of bacillus Calmette-Guerin is a loss of secreted lytic function required for invasion of lung interstitial tissue. Proc Natl Acad Sci U S A.

[14] Herr HW, Morales A (2008). History of bacillus Calmette-Guerin and bladder cancer: an immunotherapy success story. J Urol.

[15] de Martino M (2019). Immune response to mycobacterium tuberculosis: a narrative review. Front Pediatr.

[16] Harding CV (2010). Regulation of antigen presentation by Mycobacterium tuberculosis: a role for Toll-like receptors. Nat Rev Microbiol.

[17] Bonecini-Almeida MG (2004). Down-modulation of lung immune responses by interleukin-10 and transforming growth factor beta (TGF-beta) and analysis of TGF-beta receptors I and II in active tuberculosis. Infect Immun.

[18] Lombardi A (2021). T-cell exhaustion in *Mycobacterium tuberculosis* and nontuberculous mycobacteria infection: pathophysiology and therapeutic perspectives. Microorganisms.

[19] Kates M (2020). Adaptive immune resistance to intravesical BCG in non-muscle invasive bladder cancer: implications for prospective BCG-unresponsive trials. Clin Cancer Res.

[20] Wright KM (2020). FDA approves pembrolizumab for BCG-unresponsive NMIBC. Oncology (Williston Park).

[21] Shore ND (2025). Sasanlimab plus BCG in BCG-naive, high-risk non-muscle invasive bladder cancer: the randomized phase 3 CREST trial. Nat Med.

[22] Scheiber A (2024). Profiling low-mRNA content cells in complex human tissues using BD Rhapsody single-cell analysis. STAR Protoc.

[23] Lyadova IV, Panteleev AV (2015). Th1 and Th17 cells in tuberculosis: protection, pathology, and biomarkers. Mediators Inflamm.

[24] Afzali B (2007). The role of T helper 17 (Th17) and regulatory T cells (Treg) in human organ transplantation and autoimmune disease. Clin Exp Immunol.

[25] Chi LJ (2010). Involvement of T helper type 17 and regulatory T cell activity in tumour immunology of bladder carcinoma. Clin Exp Immunol.

[26] Zander R (2019). CD4^+^ T cell help is required for the formation of a cytolytic CD8^+^ T cell subset that protects against chronic infection and cancer. Immunity.

[27] Kasmani MY (2023). Clonal lineage tracing reveals mechanisms skewing CD8+ T cell fate decisions in chronic infection. J Exp Med.

[28] Shan Q (2021). Ectopic Tcf1 expression instills a stem-like program in exhausted CD8^+^ T cells to enhance viral and tumor immunity. Cell Mol Immunol.

[29] Connolly KA (2021). A reservoir of stem-like CD8 + T cells in the tumor-draining lymph node preserves the ongoing antitumor immune response. Sci Immunol.

[30] Im SJ (2016). Defining CD8+ T cells that provide the proliferative burst after PD-1 therapy. Nature.

[31] Rajaram MVS (2014). Macrophage immunoregulatory pathways in tuberculosis. Semin Immunol.

[32] Maphasa RE (2021). The macrophage response to mycobacterium tuberculosis and opportunities for autophagy inducing nanomedicines for tuberculosis therapy. Front Cell Infect Microbiol.

[33] Italiani P, Boraschi D (2014). From monocytes to M1/M2 macrophages: phenotypical vs. functional differentiation. Front Immunol.

[34] Cutolo M (2022). The role of M1/M2 macrophage polarization in rheumatoid arthritis synovitis. Front Immunol.

[35] Abdalla HB (2020). Activation of PPAR-γ induces macrophage polarization and reduces neutrophil migration mediated by heme oxygenase 1. Int Immunopharmacol.

[36] Zhang F (2016). TGF-β induces M2-like macrophage polarization via SNAIL-mediated suppression of a pro-inflammatory phenotype. Oncotarget.

[37] Waibl Polania J (2025). Antigen presentation by tumor-associated macrophages drives T cells from a progenitor exhaustion state to terminal exhaustion. Immunity.

[38] Peranzoni E (2018). Macrophages impede CD8 T cells from reaching tumor cells and limit the efficacy of anti-PD-1 treatment. Proc Natl Acad Sci U S A.

[39] Bakhru P (2014). BCG vaccine mediated reduction in the MHC-II expression of macrophages and dendritic cells is reversed by activation of Toll-like receptors 7 and 9. Cell Immunol.

[40] Meghani K (2024). Genomic and transcriptomic profiling of high-risk bladder cancer reveals diverse molecular and microenvironment ecosystems. Eur Urol.

[41] Crawford A (2011). A role for the chemokine RANTES in regulating CD8 T cell responses during chronic viral infection. PLoS Pathog.

[42] Pinhasi A, Yizhak K (2025). Uncovering gene and cellular signatures of immune checkpoint response via machine learning and single-cell RNA-seq. NPJ Precis Oncol.

[43] Juric I (2025). Single-cell RNA-sequencing of BCG naïve and recurrent non-muscle invasive bladder cancer reveals a CD6/ALCAM-mediated immune-suppressive pathway. NPJ Precis Oncol.

[44] Kelley DR (1986). Prognostic value of purified protein derivative skin test and granuloma formation in patients treated with intravesical bacillus Calmette-Guerin. J Urol.

[45] Jallad S (2014). Prognostic value of inflammation or granuloma after intravesival BCG in non-muscle-invasive bladder cancer. BJU Int.

[46] Leblond MM (2021). Tumor-associated macrophages in bladder cancer: biological role, impact on therapeutic response and perspectives for immunotherapy. Cancers (Basel).

[47] Huang Z (2015). Mycobacterium tuberculosis-induced polarization of human macrophage orchestrates the formation and development of tuberculous granulomas in vitro. PLoS One.

[48] Daman AW (2025). Microbial cancer immunotherapy reprograms hematopoiesis to enhance myeloid-driven anti-tumor immunity. Cancer Cell.

[49] Jurado LF (2025). A fungal-derived adjuvant amplifies the antitumoral potency of Bacillus Calmette-Guérin via reprogramming granulopoiesis. Immunity.

[51] Pagán AJ, Ramakrishnan L (2018). The formation and function of granulomas. Annu Rev Immunol.

[52] Cooper AM (2009). Cell-mediated immune responses in tuberculosis. Annu Rev Immunol.

[53] Kim S (2022). Mycobacterium intracellulare induces a Th17 immune response via M1-like macrophage polarization in canine peripheral blood mononuclear cells. Sci Rep.

[54] Liu J (2018). Expression of IL-23R and IL-17 and the pathology and prognosis of urinary bladder carcinoma. Oncol Lett.

[55] Takeuchi A (2011). IL-17 production by γδ T cells is important for the antitumor effect of Mycobacterium bovis bacillus Calmette-Guérin treatment against bladder cancer. Eur J Immunol.

[56] Andrade PR (2025). Dynamics of Th1/Th17 responses and antimicrobial pathways in leprosy skin lesions. J Clin Invest.

[57] Lázár-Molnár E (2010). Programmed death-1 (PD-1)-deficient mice are extraordinarily sensitive to tuberculosis. Proc Natl Acad Sci U S A.

[58] Kauffman KD (2021). PD-1 blockade exacerbates Mycobacterium tuberculosis infection in rhesus macaques. Sci Immunol.

[59] Barber DL (2019). Tuberculosis following PD-1 blockade for cancer immunotherapy. Sci Transl Med.

[60] Tzelepis F (2018). Mitochondrial cyclophilin D regulates T cell metabolic responses and disease tolerance to tuberculosis. Sci Immunol.

[61] Tameris MD (2013). Safety and efficacy of MVA85A, a new tuberculosis vaccine, in infants previously vaccinated with BCG: a randomised, placebo-controlled phase 2b trial. Lancet.

[62] Nemes E (2018). Prevention of M. tuberculosis Infection with H4:IC31 Vaccine or BCG Revaccination. N Engl J Med.

