# Supplementary material for: Divergent macrophage-regulated T cell states determine response to Bacillus Calmette-Guérin vaccine in high-risk bladder cancer
Source: J Clin Invest. 2026 Apr 21;136(12):e200442. doi: 10.1172/JCI200442 (PMC13262731; doi:10.1172/JCI200442)
Supplement: Supplemental data [file jci-136-200442-s049.pdf]

Supplementary Methods

Sample Processing

Tissue was transported in DMEM with 10% FBS to preserve stability at room temperature to preserve temperature sensitive granulocytes. Tissue was minced and digested in two rounds (20 minutes and 15 minutes) in DMEM without FBS using Liberase TM (Sigma) in a 37 °C water bath with gentle manual inversion. The digested tissue was resuspended in FACs buffer passed through a 70 uM strainer (Corning), centrifuged and subjected to CD45 enrichment using the Miltenyi biotech CD45 microbeads as per the manufacturer's instructions.

In parallel, 1 ml of blood from blood isolated in EDTA coated tubes was also processed to isolate leukocytes using whole blood lysis protocol recommended by 10X Genomics (CG000392) using eBioscience 1X RBC Lysis Buffer (Thermo fisher) with PBS washes and low speed centrifugation.

BD Rhapsody single-cell RNA sequencing

To combine the matched tissue and blood samples to load on a single BD Rhapsody cartridge, the processed matched tissue and blood samples were labeled with sample tags using the BD Single-cell Multiplexing kit in accordance with the manufacturer's protocol. The individual samples were resuspended in BD Rhapsody sample buffer (BD Biosciences) and stained with DRAQ7 and Calcein AM florescent dyes to enable visualization for counting and calculation of quality control metrics in the BD Rhapsody single cell system. The cells were counted, and blood-tissue samples mixed, passed through a 70 micron strainer and up to 10,000 cells loaded in the BD Rhapsody microwell cartridge system. Magnetic capture beads were then loaded close to saturation so that each microwell contains one magnetic bead. Each of the cells were isolated in the microwells, lysed and the polyadenylated mRNA molecules from each cell captured by individual magnetic capture beads containing unique molecular identifiers (UMIs) and cell specific barcodes. The capture beads were retrieved from the BD Rhapsody single cell system and cDNA reverse transcription carried out with cDNA with UMI and cell specific barcode information added to the cDNA molecules. 3 'end whole transcriptome amplification (WTA), sample tag libraries were prepared according to the BD Rhapsody WTA and sample tag preparation protocol. For quantification and quality control purposes the libraries were subjected to Qubit and Tape station analyses. For calculation of total paired end reads, sequencing depth of 60,000 reads per cell

were used for the WTA library analyses, for the sample tag libraries sequencing depth 1200 reads per cell. The WTA and sample tag libraries were mixed according to custom BD Rhapsody calculations. The libraries were subjected to sequencing using 150 bp paired end sequencing using the Novaseq X Plus by Admera Health (New Jersey).

Data Processing

The BD Rhapsody fastq files were processed using the BD Rhapsody WTA pipeline, available on the Seven Bridges Genomics cloud server. Briefly the pipeline had steps which involved filtering of the reads, identifying the cell ID and the UMI, aligning the reads to the the hg38 reference genome or in the case of Abseq data, the Abseq reference file. The raw counts were error corrected, putative cells identified and the final processed file contained reads per cell for the samples generated along with the quality control metrics.

Data filtering and quality control

The Seurat objects generated at the end of the BD Rhapsody WTA pipeline were downloaded and further processing was done using Seurat (v3) in R version 4.3.2/4.3.3 (63–65). Initially quality control filtering was carried out to remove low expressing cells or potential doublets as recommended by the Seurat pipeline removing cells with high percentage of mitochondrial reads and low expressing cells. Cells identified as having no sample tags ("Undetermined") or identified as multiplets by the BD Rhapsody pipeline were also removed. To ensure that singlets were identified, DoubletFinder was run on each sample to remove doublets (66).

Data Analysis

All analyses were performed in R. Integration across all samples was performed with canonical correlation analysis. Analysis of cellular subsets were reintegrated using either SCTransform, CCA, or harmony. Determination of integration method was based on visualized batch effects on the umap plot. Data visualization was carried out using the ggpubr and ggplot2 packages. Box-and-whisker plots were generated with ggboxplot() specifying sample groups as factors, and statistical comparisons were overlaid using the stat_compare_means() function with appropriate tests (t test, anova, or Kruskal–Wallis, depending on distribution). For box plots where percentages were calculated in the tissue compartment, a minimum cell threshold was set at 100 cells per patient

to be included and the cell threshold would be reduced by increments of 25 cells until each BCG condition had at least 3 samples. Additional visualizations including stacked bar plots were generated with ggplot() using customized themes.

Trajectory inference was performed using the Slingshot package (67). Input data consisted of dimensionally reduced coordinates from Seurat UMAP embeddings, along with cluster labels. Slingshot was run with default parameters, initializing trajectories from the earliest cell cluster, and pseudotime values were assigned to each cell based on principal curves fitted through the low-dimensional space.

For heatmap generation, we first identified differential expression between clusters through Seurat's FindAllMarkers() function. Genes meeting significance thresholds (adjusted $p < 0.05$) were selected and visualized with the ComplexHeatmap package (68). Heatmaps were constructed with Heatmap() using row scaling (scale = "row") and Euclidean distance–based clustering. The number of cells per patient were down-sampled to remove differences in sequencing depth across patients. Hierarchical clustering was initialized at the level of the number of predefined cell types, and the tree was iteratively cut into higher numbers of clusters until additional subdivisions resulted in redundant or overlapping gene modules.

Cell–cell communication was inferred with CellChat (69). All cells from the bladder samples were included as input to createCellChat(). Normalization and preprocessing followed the CellChat workflow. Networks were inferred with computeCommunProb() using default parameters, and aggregated at the signaling pathway level with computeCommunProbPathway(). Communication probability matrices were then projected into signaling networks for visualization.

Machine learning analysis was conducted with the Precise package using the complete bladder dataset as input. Gene selection was first performed using the Boruta algorithm. The resulting gene set was used to train an XGBoost model. The final model was integrated with Precise's reinforcement learning framework, which iteratively updates predictive weights based on misclassified cells. Each cell was assigned a score corresponding to its predicted class probability, which was used in downstream analyses.

Validation of gene signatures was performed on previously published bulk RNA-seq study of 103 BCG-treated patients. For each sample, a composite expression score was calculated as the mean log-transformed expression across the IL17A/IL21/IL26 genes. The mean of averaged expression values across patients was selected as the cutoff point. All samples with averaged expression higher than this cutoff were assigned as high expression, with all samples with expression lower than this cutoff were assigned as low expression.

Formalin-fixed paraffin embedded (FFPE) slide preparation

Selected FFPE blocks were identified from the previously enrolled patients. There was an equal number of BCG responsive (n=3) and BCG unresponsive (n=3) samples selected based on tissue size and the presence of normal adjacent, non-tumor, bladder tissue. Blocks were sectioned using a microtome onto a positively charged slide (PN# 1255015; Fisherband) at a thickness of 5um. Directly adjacent sections were prepared and subsequently stained using hematoxylin and eosin (H&E) for representative tissue region identification. Unstained tissue slides were allowed to dry overnight and used for the following multiplex immunofluorescence assay described below.

Multiplex immunofluorescence sample processing

Slides were baked for 1 h at 60C on a slide warmer and dewaxed through two successive rounds of incubation in Histochoice Clearing Agent (PN# H103-4L; VWR) for 5 min each. Subsequently, the FFPE sections were dehydrated by sequentially incubating in 100% ethanol (PN# 79317-16GA-PB; Sigma Aldrich), 90% ethanol, 70% ethanol, 50% ethanol, and 30% ethanol twice each for 5 min. Slides were then rinsed three times in distilled water for 5 min to ensure no carryover of ethanol before target retrieval. Heat-mediated epitope retrieval was performed by incubating slides in a beaker containing Tris EDTA solution (pH 9; Akoya Biosciences) in a pressure cooker for 20 min. After 20 min, the beaker was removed from the pressure cooker and left to equilibrate at room temperature for 30 min. Slides were then rinsed through two rounds of incubation in distilled water for 2 min and stored in Hydration Buffer from the Sample Kit for PhenoCycler- Fusion (#7000017; Akoya Biosciences) until ready for staining. For antibody staining, an antibody cocktail was prepared with optimal dilutions of each antibody in a buffer containing N, J, S and G blockers (#7000017;

Akoya Biosciences). Samples were first allowed to equilibrate to room temperature in Staining Buffer for 20–30 min, followed by incubation in a pre-blocking solution made of N, J and S blockers in Staining Buffer. The antibody cocktail was subsequently added to the slide, and slides were incubated for 3 hours at room temperature. After washing with staining buffer and fixation in 1.6% PFA, samples were fixed in ice-cold methanol for 5 min at 4C. A third round of fixation with PhenoCycler Fixative reagent was applied, and slides were stored in Storage Buffer at 4C until ready to image.

Multiplex immunofluorescence reporter plate preparation and imaging

Reporter Stock solution was prepared as described in the PhenoCycler-Fusion User Guide with 10 X Buffer (#7000019), Assay Reagent (#7000002), and Nuclear Stain (#7000003) from Akoya Biosciences. Individual tubes of 3 reporters/ cycle, diluted in reporter stock solution, were prepared. Subsequently, a 96- well plate was prepared with 1-well/cycle containing the corresponding working reporter solution for that cycle. Blank cycles containing only reporter stock solution without any added fluorophore reporters were included as the first and last cycle in each run for subtraction of autofluorescence background. Exact sequence of cycles, reporter and barcode information can be found in (Table S1). Antibody-stained slides were next equilibrated at room temperature in 1X Buffer for at least 10 min. The flow cell (#240204; Akoya Biosciences) was assembled onto the slide as described in the user guide. The slides were incubated in Buffer again for 10 min to ensure secure sealing of the flow cell to the slide. The slide was then transferred to the flow cell carrier and imaged on a PhenoCycler-Fusion (Akoya Biosciences) using the following exposure settings: DAPI—1 ms, ATTO550 channel—150 ms, AF647 channel— 150 ms and AF750 channel—150 ms. Integration of the PhenoCycler automated fluidics cycler with the PhenoImager Fusion imaging system automated the entire process of reporter hybridization, imaging, and dehybridization to capture whole slide images of three markers (+DAPI for nuclear staining) at a time. After images of all cycles had been acquired, the final QPTIFF file containing a composite image of all markers was exported.

Nuclei segmentation and pixel measurements

Exported QPTIFF images were uploaded and processed using QuPath v0.6.0, an image processing software (70), with the additional StarDist extension added for nuclei segmentation (71). Each slide contained two

samples. Regions of interest were selected on each sample verified to be free of extensive cautery artifacts, necrosis, and large tumor regions. These regions were identified by a pathologist, B.C., from the corresponding adjacent H&E tissue image and selected by A.H. on the sample-matched immunofluorescence image. Once regions were selected, a custom script, cell_detection, was implemented to utilize a trained model with the DAPI channel and set detection parameters.

Cell type classification

A representative tissue sample region with marked expression of all markers was chosen for classification. QuPath single measurement classifier was utilized to determine set thresholds of signal positivity. Staining was verified across regions within the tissue. Cell mean pixel intensity was used for a given marker based on expected localization pattern. Threshold intensity values for all markers included in classification can be found in the cell_type_classification script. Set intensity thresholds were used across slides and for each sample. Known canonical markers were used to assess the following cell types of interest; epithelial cells (Pan-Cytokeratin), immune cells (CD45), CD4 T cells (CD3e, CD4), CD8 T cells (CD3e, CD8), macrophages (CD14/CD68, HLA-A/HLA-DR), TAM (CD14/CD68, CD163, CD206), and dendritic cells (CD11c, HLA-A/HLA-DR). A representative image of P8, shown in (Supplemental Figure 9), was exported from QuPath of the composite image and individual channels. Cells were deemed positive if the mean pixel intensity of a given cell was above the set threshold intensity. A final exported table of mean pixel intensity values, positivity flag for a given marker, and final phenotype classification was used for additional processing.

Proximity calculations for distance between cell types

Once cell type classifications were generated spatial object processing was done in R v4.4.3 using the SPIAT package v1.8(72). The finalized data frame described above was used as input to generate a summarized spatial experiment object for each sample. The proximity between the cell types was determined using the calculate_minimum_distances_between_celltypes() argument in SPIAT. Density plot was generated using geom_density from the ggplot2 package. This method uses a Gaussian kernel to estimate the probability distribution and create a smoothed, continuous line. The proportion calculations were performed on the data

frame resulting from distance calculations. The percentage was determined out of the total number of reference cells for the selected cell types shown in (Supplemental Figure 9).

Supplementary Figure Legends

Supplementary Figure 1. Immune cell gene expression and distributions validation

(A) Feature plot of representative genes for each major cluster. **(B)** UMAP plot of cells in both the blood and tissue. **(C)** Stacked bar plots summarizing cell subset proportions after down sampling to 200 cells per sample in the blood tissue.

Supplementary Figure 2. Distribution of immune cell types across blood and tissue

(A) Stacked bar plot summarizing cell count (top) and cell proportions (bottom) for each patient's bladder. **(B)** Stacked bar plot summarizing cell count (top) and cell proportions (bottom) for each patient's blood.

Supplementary Figure 3. Cytotoxic and effector NK cell signatures are enriched in BCG responders

(A) UMAP of subset NK cells from the blood and bladder tissue. **(B)** Dot plot of representative genes expressed in each major cluster. Dot size represents percent of cells expressing the gene; color represents scaled expression of the gene. **(C)** Slingshot trajectory of NK T cells. **(D)** Correlation between a patient's proportion of CD56-dim, CD16+ cells and TrNK cells. **(E)** Proportion of NK cell subsets in blood and bladder. Box plots showing the distribution of immune cell type proportions for each patient. **(F)** Proportion NK cell subsets in BCG naïve, responsive, and unresponsive groups in the bladder tissue. Stacked bar plots summarizing NK cell subset proportions after down sampling to 50 cells per sample. **(G)** Proportion of NK cell subsets in BCG naïve, responsive and unresponsive groups in the bladder tissue. **(H)** Proportion of NK cell subsets in BCG naïve, responsive and unresponsive groups in the blood.

Supplementary Figure 4. CD4+ T cell composition in blood and bladder

(A) Cosine similarity scores between subsets of CD4+ T cells. **(B)** UMAP of blood and bladder tissue cells. **(C)** Proportion of CD4+ cell subsets in BCG naïve, responsive and unresponsive groups in the blood.

Supplementary Figure 5. CD8+ T cell composition in blood and bladder

(A) Feature plot of representative genes for each major cluster. **(B)** UMAP plot of cells in both the blood and tissue. **(C)** Stacked bar plots summarizing cell subset proportions after down sampling to 50 cells per sample in the blood tissue. **(D)** Flow cytometry of CD8 T cells from a separate cohort of BCG treated tumors

Supplementary Figure 6. BD-Rhapsody identifies cellular diversity of neutrophils responding to bladder cancer.

(A) UMAP of neutrophils from the blood **(B)** UMAP of neutrophils from the bladder tissue. **(C)** Dot plot of representative genes expressed in each major cluster. Dot size represents percent of cells expressing the gene; color represents scaled expression of the gene. **(D)** Proportion of neutrophils cell subsets in blood and bladder. Box plots showing the distribution of immune cell type proportions for each patient. **(E)** Proportion of neutrophils cell subsets in BCG naïve, responsive and unresponsive groups in the blood. **(F)** Proportion of neutrophils cell subsets in BCG naïve, responsive and unresponsive groups in the bladder tissue. **(G)** Proportion of neutrophils cell subsets in BCG naïve, responsive and unresponsive groups in the bladder tissue when including all other cell types. **(H)** Proportion neutrophils cell subsets in BCG naïve, responsive, and unresponsive groups in the bladder tissue. Stacked bar plots summarizing neutrophils cell subset proportions after down sampling to 50 cells per sample.

Supplementary Figure 7. Monocytes and macrophage composition in blood and bladder

(A) UMAP of monocyte and macrophage subsets between the blood and bladder tissue. **(B)** Proportion of monocyte and macrophage cell subsets in BCG naïve, responsive and unresponsive groups in the blood. **(C)** Proportion of monocyte and macrophage cell subsets in BCG naïve, responsive and unresponsive groups in the bladder tissue.

Supplementary Figure 8. CD4+ T cell interactions define responders

(A) Dot Plot of MHCII cellular interactions between antigen presenting cells and CD4+ T cells. **(B)** Survival curve of CD74 and HLA-DOA over the course of two years. **(C)** Stacked bar plot of signaling found between CD4+ T cells and CD8+ T cells in BCG responders and non-responders (left). Expression of CCL5 in CD4+ T cells (middle) and CCR5 in CD8+ T cells (right).

Supplementary Figure 9: Cell-cell proximity resolved by multiplex immunofluorescence

(A) Spatial tissue distribution of classified cell types based on protein marker expression from a representative BCG responsive sample; P8. Marker classification is described in detail in methods, see cell type classification. Spatial image coordinates are plotted and the corresponding cell type for each given cell is displayed across the tissue. **(B)** Spatial tissue distribution of classified cell types based on protein marker expression from a representative BCG unresponsive sample; P14. Marker classification is described in detail in methods, see cell type classification. Spatial image coordinates are plotted and the corresponding cell type for each given cell is displayed across the tissue. **(C)** Representative fluorescence image from the indicated outlined regions from sample P8 displayed in panel A. The first image displays the composite overlay of the following channels CD4, CD11c, CD45, CD68, and HLA-DR to visualize interactions of CD4+ T cells, antigen presenting dendritic cells and macrophages. Individual channel images are displayed below from the same region to display single-marker signal intensity. DAPI in gray marks individual nuclei, CD4 in yellow for CD4+ T cells, CD45 in blue is a cell surface receptor expressed on general immune cells, CD68 in green is expressed by macrophages, CD11c in maroon is a cell surface marker of dendritic cells, and HLA-DR in pink is the class II surface receptor, expressed by macrophages and dendritic cells for presentation to CD4+ T cells. **(D)** Distance calculations determine the proximity of the reference cell type, dendritic cells (DCs), to the nearest cell type, CD4+ T cells. Density plot (left) approximates the distribution, see methods, based on the number of CD4+ T cells within 50 µm of DCs (n = 6). Boxplot (right) quantifies the percentage of DCs out of the total number of DCs for each sample were within 50 µm and compares the distribution between BCG responsive and unresponsive groups (n = 6). **(E)** Distance calculations determine the proximity of the reference cell type, tumor associated macrophages (TAMs), to the nearest cell type, CD4+ T cells. Density plot (left) approximates the distribution, see methods, based on the number of CD4+ T cells within 50 µm of TAMs (n = 5). P12 sample is excluded, due to less than 2 TAM cells within 50 µm, unable to estimate density. Boxplot (right) quantifies the percentage of TAMs out of the total number of TAMs for each sample were within 50 µm and compares the distribution between BCG responsive and unresponsive groups (n = 6).

Supplementary Figure 10. Machine learning identifies responder and non-responder gene sets.

(A) Dot Plot of Boruta selected genes grouped by cell type expression**. (B)** SHAP value scores for Boruta selected genes.

Supplementary Methods References

62. Nemes E, Geldenhuys H, Rozot V, Rutkowski KT, Ratangee F, Bilek N, et al. Prevention of M. tuberculosis Infection with H4:IC31 Vaccine or BCG Revaccination. N Engl J Med. 2018 Jul 12;379(2):138–49.
63. Butler A, Hoffman P, Smibert P, Papalexi E, Satija R. Integrating single-cell transcriptomic data across different conditions, technologies, and species. Nat Biotechnol. 2018 May 2;36(5):411–20.
64. Stuart T, Butler A, Hoffman P, Hafemeister C, Papalexi E, Mauck WM, et al. Comprehensive Integration of Single-Cell Data. Cell. 2019 Jun;177(7):1888-1902.e21.
65. Hao Y, Hao S, Andersen-Nissen E, Mauck WM, Zheng S, Butler A, et al. Integrated analysis of multimodal single-cell data. Cell. 2021 Jun;184(13):3573-3587.e29.
66. McGinnis CS, Murrow LM, Gartner ZJ. DoubletFinder: Doublet Detection in Single-Cell RNA Sequencing Data Using Artificial Nearest Neighbors. Cell Syst. 2019 Apr;8(4):329-337.e4.
67. Street K, Risso D, Fletcher RB, Das D, Ngai J, Yosef N, et al. Slingshot: cell lineage and pseudotime inference for single-cell transcriptomics. BMC Genomics. 2018 Dec 19;19(1):477.
68. Gu Z, Eils R, Schlesner M. Complex heatmaps reveal patterns and correlations in multidimensional genomic data. Bioinformatics. 2016 Sep 15;32(18):2847–9.
69. Jin S, Guerrero-Juarez CF, Zhang L, Chang I, Ramos R, Kuan CH, et al. Inference and analysis of cell-cell communication using CellChat. Nat Commun. 2021 Feb 17;12(1):1088.
70. Bankhead P, Loughrey MB, Fernández JA, Dombrowski Y, McArt DG, Dunne PD, et al. QuPath: Open source software for digital pathology image analysis. Sci Rep. 2017 Dec 4;7(1):16878.
71. Schmidt U, Weigert M, Broaddus C, Myers G. Cell Detection with Star-convex Polygons. 2018 Nov 8;
72. Feng Y, Yang T, Zhu J, Li M, Doyle M, Ozcoban V, et al. Spatial analysis with SPIAT and spaSim to characterize and simulate tissue microenvironments. Nat Commun. 2023 May 15;14(1):2697.

Figure S1

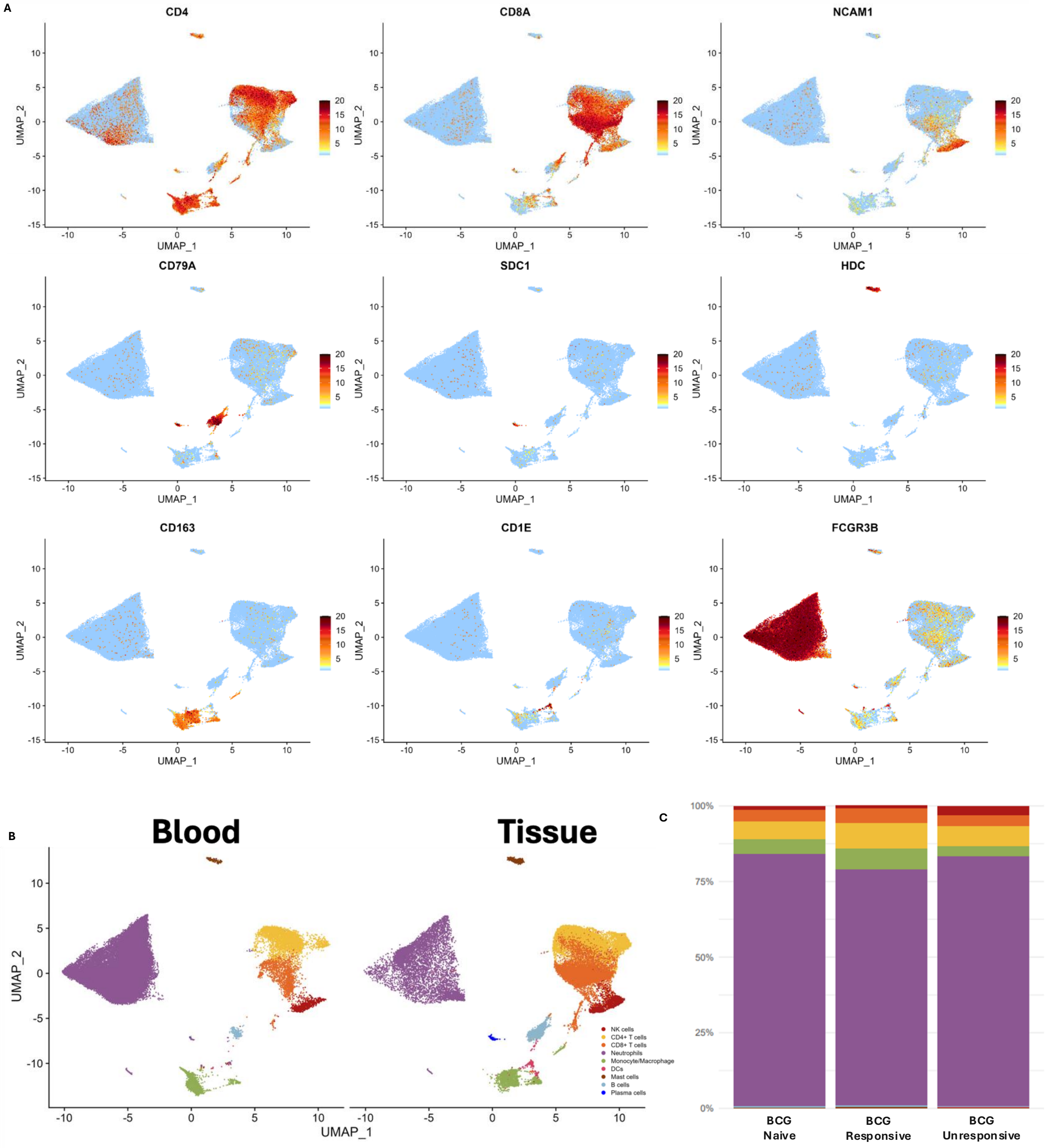
A
CD4
CD8A
NCAM1
CD79A
SDC1
HDC
CD163
CD1E
FCGR3B
UMAP_1
UMAP_2
B
Blood
Tissue
NK cells
CD4+ T cells
CD8+ T cells
Neutrophils
Monocyte/Macrophage
DCs
Mast cells
B cells
Plasma cells
C
100%
75%
50%
25%
0%
BCG Naive
BCG Responsive
BCG Unresponsive

Figure S2

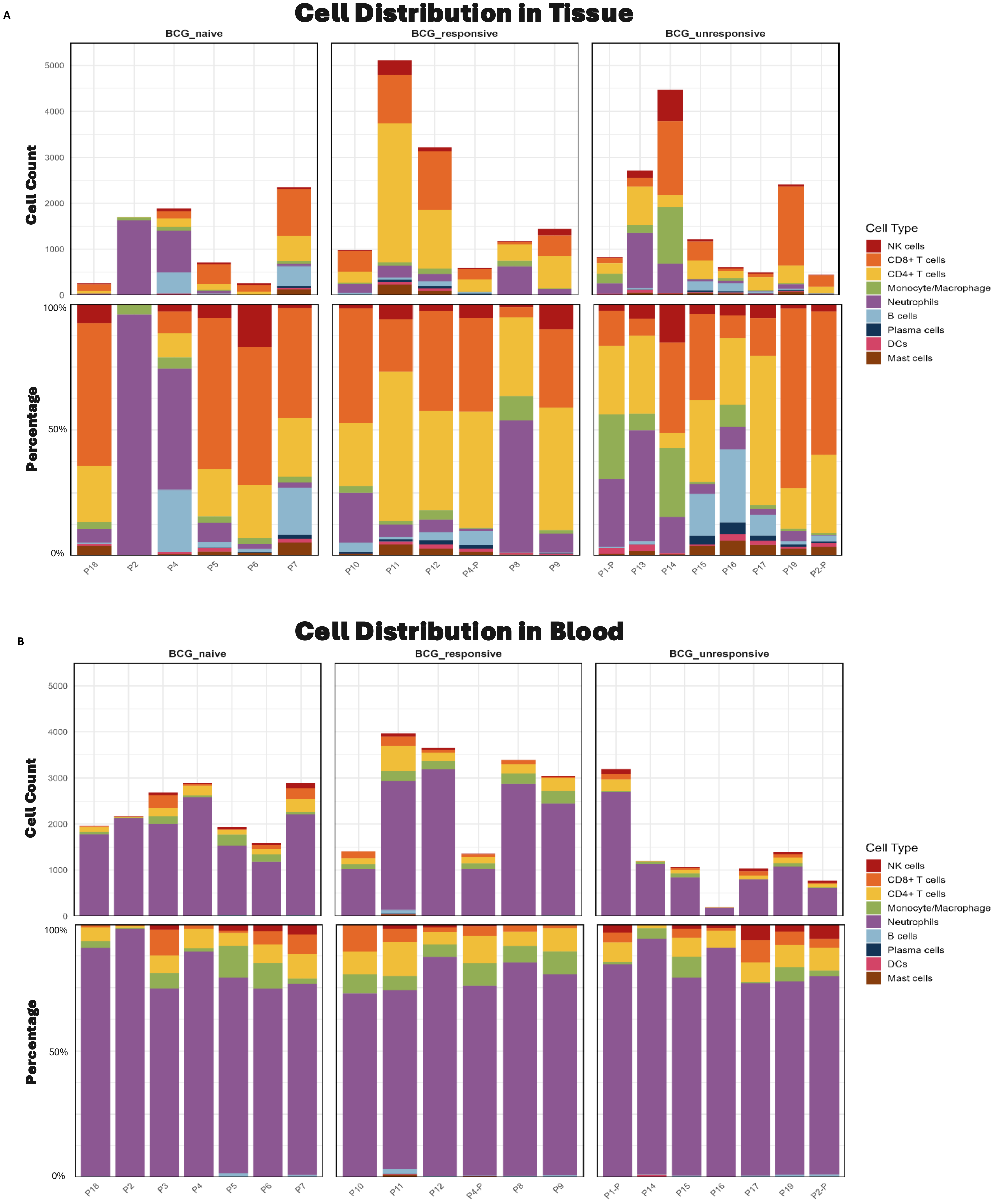
A
Cell Distribution in Tissue
BCG_naive
BCG_responsive
BCG_unresponsive
Cell Count
Percentage
0
1000
2000
3000
4000
5000
0%
50%
100%
P18 P2 P4 P5 P6 P7
P10 P11 P12 P4-P P8 P9
P1-P P13 P14 P15 P16 P17 P19 P2-P
Cell Type
NK cells
CD8+ T cells
CD4+ T cells
Monocyte/Macrophage
Neutrophils
B cells
Plasma cells
DCs
Mast cells
B
Cell Distribution in Blood
BCG_naive
BCG_responsive
BCG_unresponsive
Cell Count
Percentage
0
1000
2000
3000
4000
5000
0%
50%
100%
P18 P2 P3 P4 P5 P6 P7
P10 P11 P12 P4-P P8 P9
P1-P P14 P15 P16 P17 P19 P2-P
Cell Type
NK cells
CD8+ T cells
CD4+ T cells
Monocyte/Macrophage
Neutrophils
B cells
Plasma cells
DCs
Mast cells

Figure S3

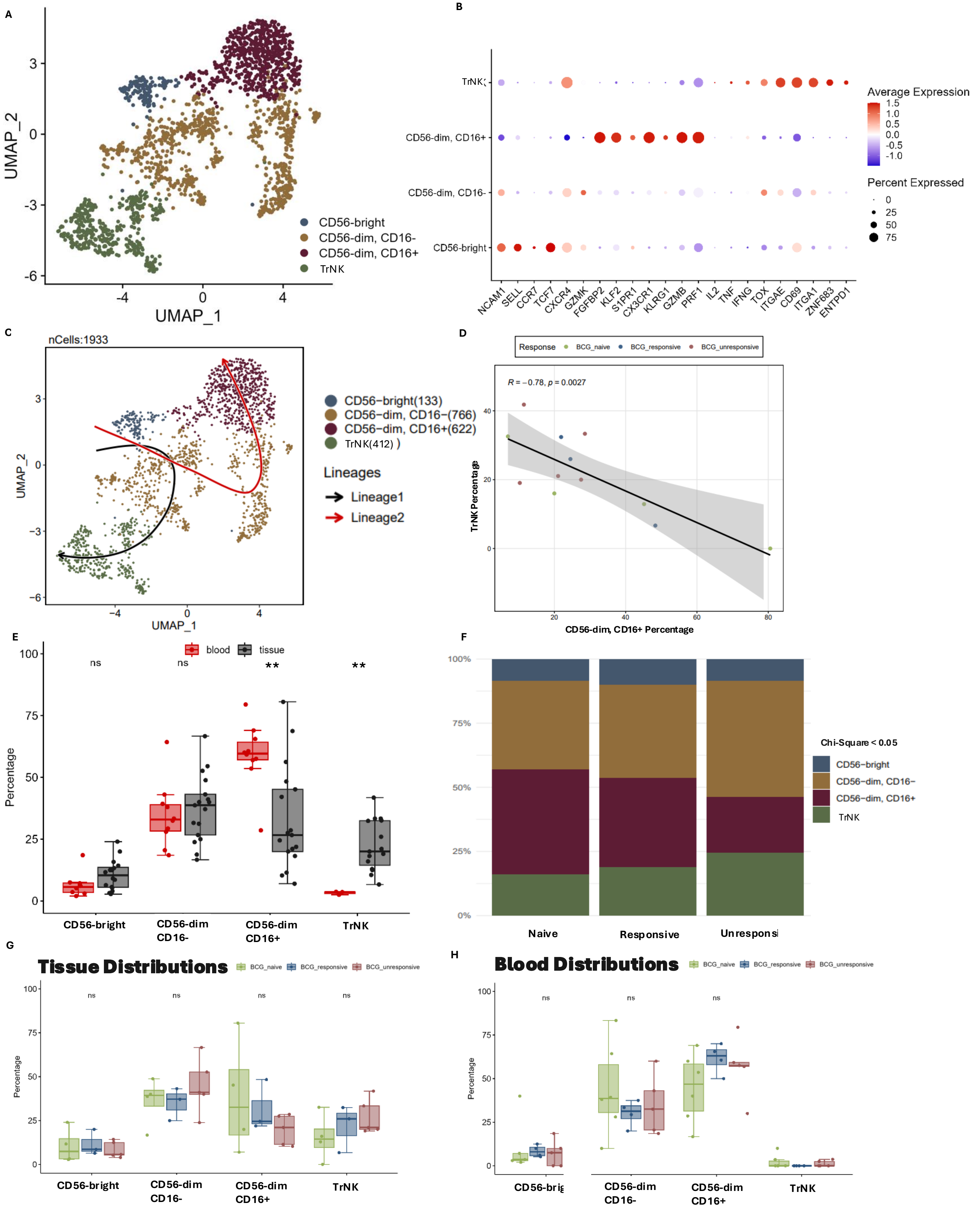

A
UMAP_1
UMAP_2
CD56-bright
CD56-dim, CD16-
CD56-dim, CD16+
TrNK
B
Average Expression
Percent Expressed
NCAM1
SELL
CCR7
TCF7
CXCR4
GZMK
FGFBP2
KLF2
S1PR1
CX3CR1
KLRG1
GZMB
PRF1
IL2
TNF
IFNG
TOX
ITGAE
CD69
ITGA1
ZNF683
ENTPD1
C
nCells:1933
CD56−bright(133)
CD56−dim, CD16−(766)
CD56−dim, CD16+(622)
TrNK(412))
Lineages
Lineage1
Lineage2
D
Response
BCG_naive
BCG_responsive
BCG_unresponsive
R = −0.78, p = 0.0027
TrNK Percentage
CD56-dim, CD16+ Percentage
E
blood
tissue
ns
ns
**
**
Percentage
CD56-bright
CD56-dim CD16-
CD56-dim CD16+
TrNK
F
Chi-Square < 0.05
CD56-bright
CD56-dim, CD16−
CD56-dim, CD16+
TrNK
Naive
Responsive
Unresponsi
G
Tissue Distributions
BCG_naive
BCG_responsive
BCG_unresponsive
ns
ns
ns
ns
Percentage
CD56-bright
CD56-dim CD16-
CD56-dim CD16+
TrNK
H
Blood Distributions
BCG_naive
BCG_responsive
BCG_unresponsive
ns
ns
ns
Percentage
CD56-brig
CD56-dim CD16-
CD56-dim CD16+
TrNK

Figure S4

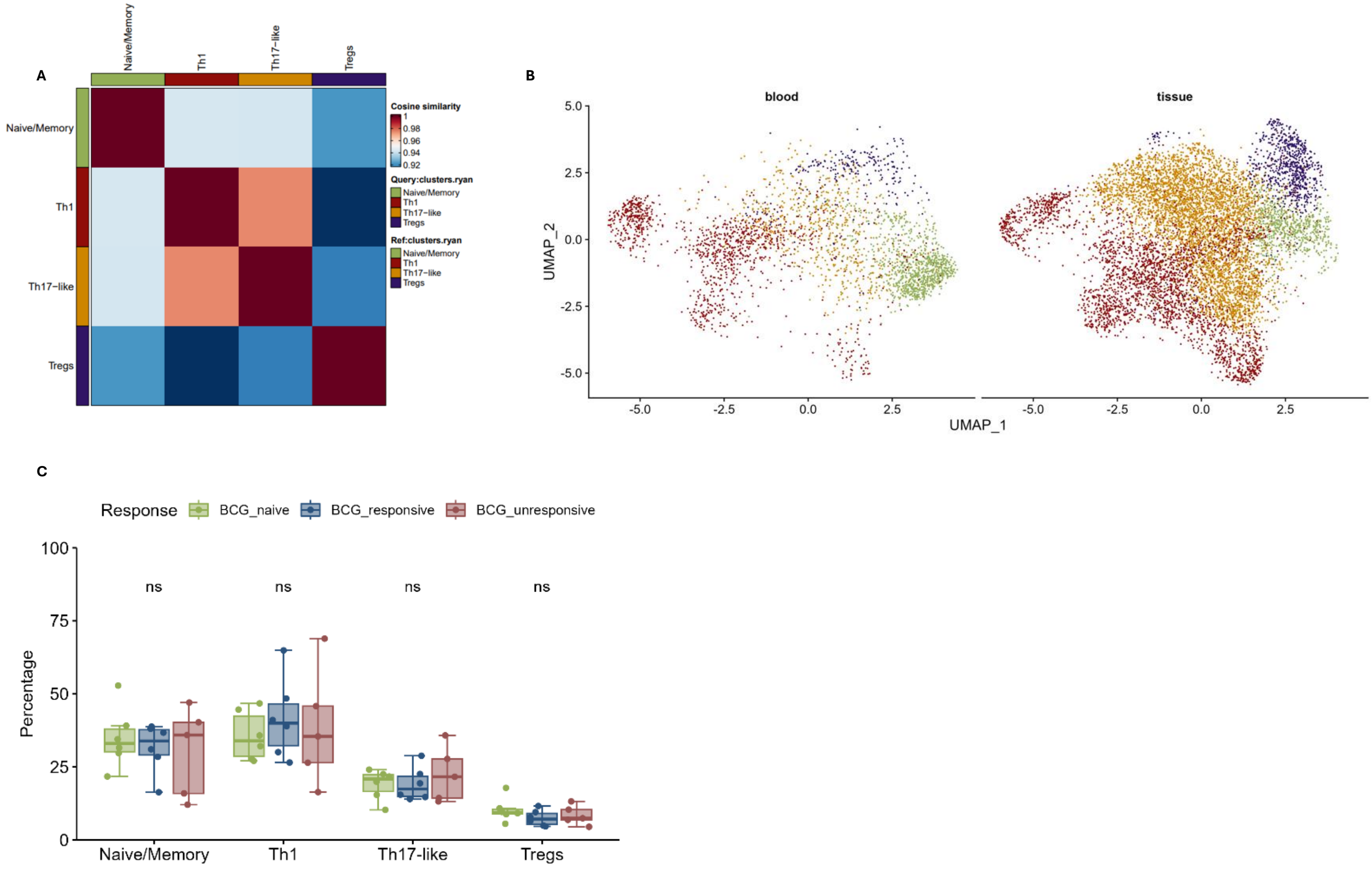
A
Naive/Memory
Th1
Th17−like
Tregs
Cosine similarity
1
0.98
0.96
0.94
0.92
Query:clusters.ryan
Naive/Memory
Th1
Th17−like
Tregs
Ref:clusters.ryan
Naive/Memory
Th1
Th17−like
Tregs
B
blood
tissue
UMAP_2
UMAP_1
5.0
2.5
0.0
-2.5
-5.0
C
Response
BCG_naive
BCG_responsive
BCG_unresponsive
Percentage
100
75
50
25
0
ns
Naive/Memory
Th1
Th17-like
Tregs

Figure S5

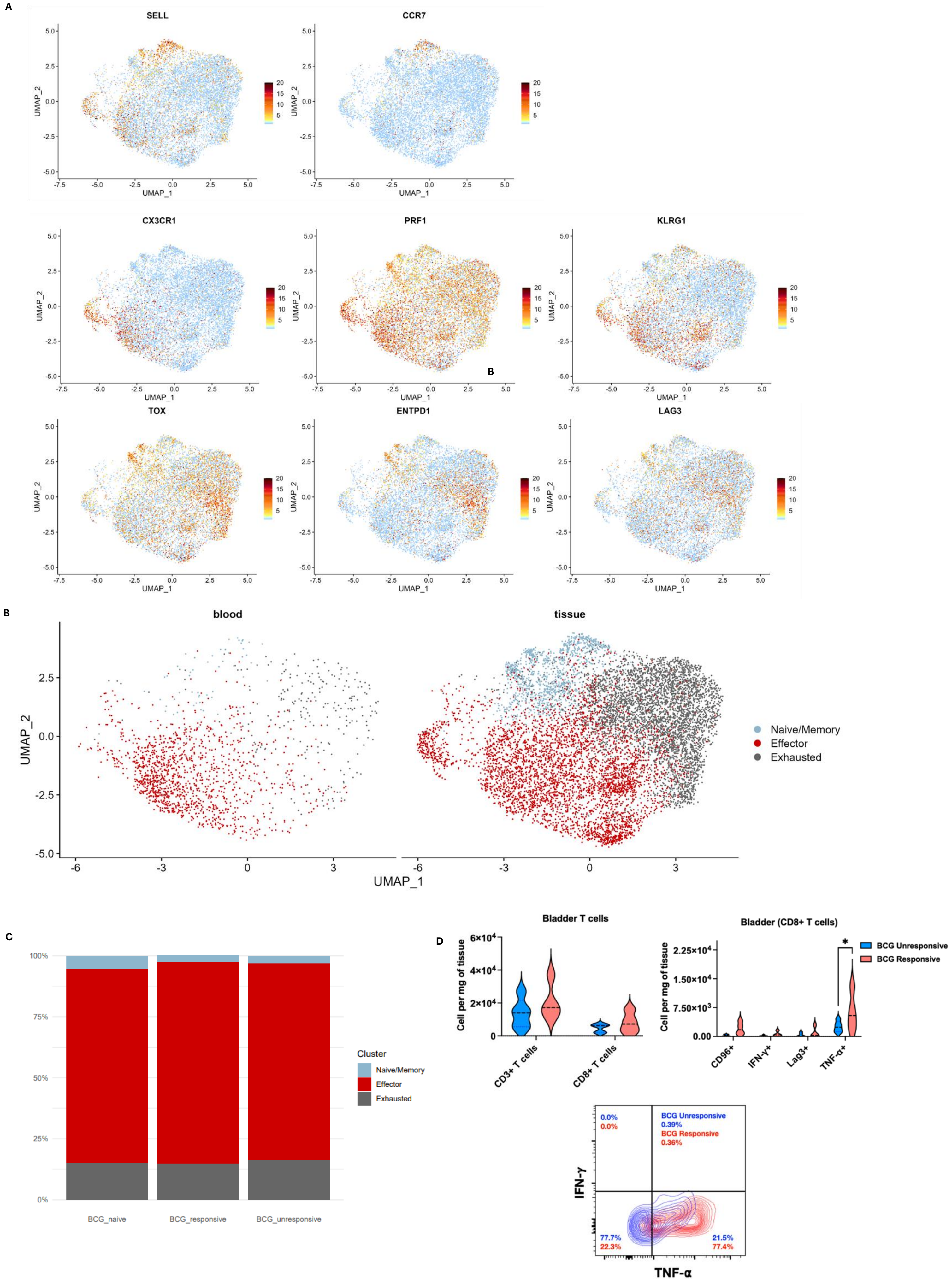
A
SELL
CCR7
CX3CR1
PRF1
KLRG1
TOX
ENTPD1
LAG3
UMAP_1
UMAP_2
B
blood
tissue
UMAP_1
UMAP_2
Naive/Memory
Effector
Exhausted
C
Cluster
Naive/Memory
Effector
Exhausted
BCG_naive
BCG_responsive
BCG_unresponsive
D
Bladder T cells
Cell per mg of tissue
CD3+ T cells
CD8+ T cells
Bladder (CD8+ T cells)
CD96+
IFN-γ+
Lag3+
TNF-α+
BCG Unresponsive
BCG Responsive
0.0%
0.0%
BCG Unresponsive
0.39%
BCG Responsive
0.36%
77.7%
22.3%
21.5%
77.4%
IFN-γ
TNF-α

Figure S6

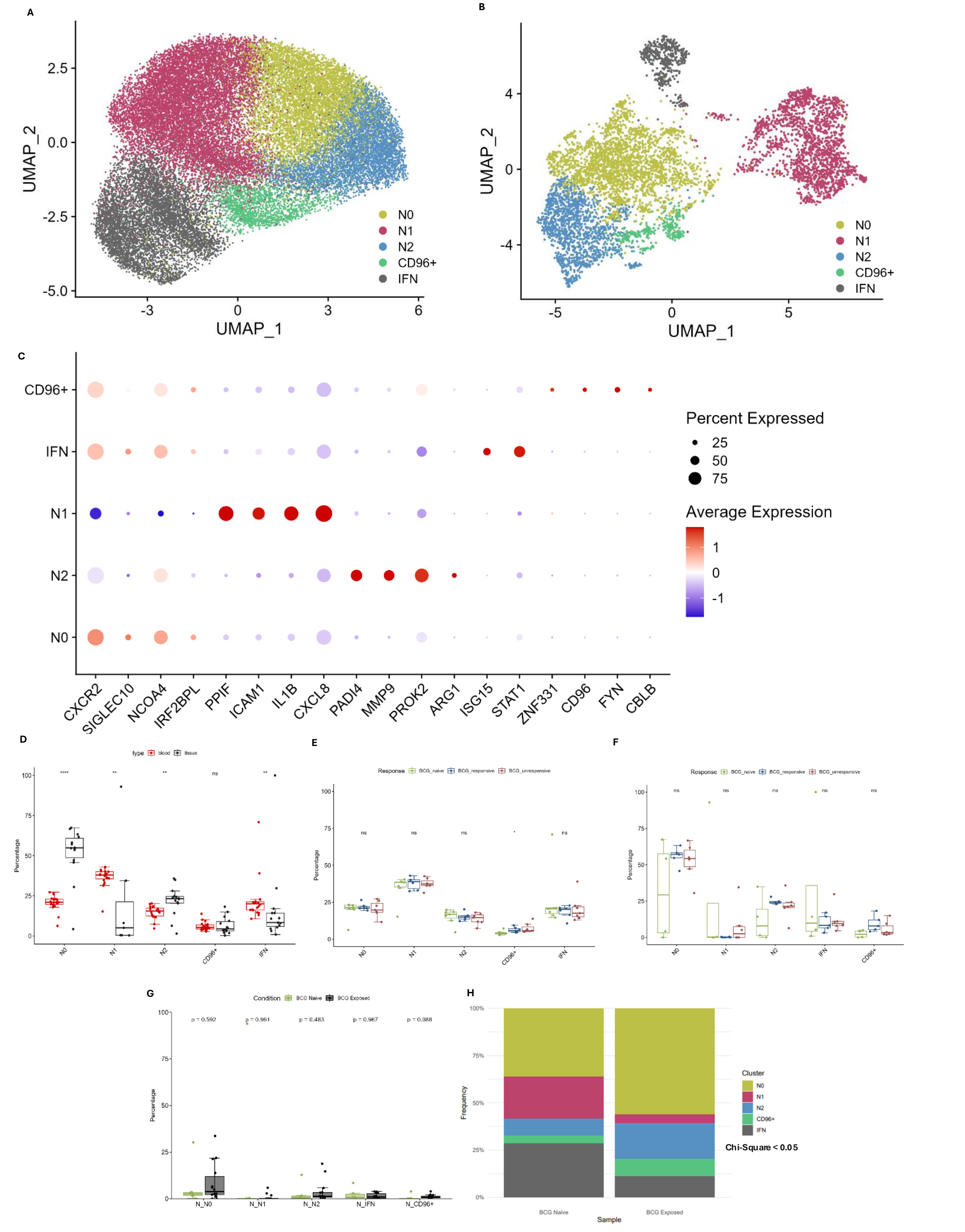
A
UMAP_2
UMAP_1
2.5
0.0
-2.5
-5.0
-3
0
3
6
N0
N1
N2
CD96+
IFN
B
UMAP_2
UMAP_1
4
0
-4
-5
0
5
N0
N1
N2
CD96+
IFN
C
CD96+
IFN
N1
N2
N0
CXCR2
SIGLEC10
NCOA4
IRF2BPL
PPIF
ICAM1
IL1B
CXCL8
PADI4
MMP9
PROK2
ARG1
ISG15
STAT1
ZNF331
CD96
FYN
CBLB
Percent Expressed
25
50
75
Average Expression
1
0
-1
D
type blood tissue
Percentage
100
75
50
25
0
****
**
**
ns
**
N0
N1
N2
CD96+
IFN
E
Response BCG_naive BCG_responsive BCG_unresponsive
Percentage
100
75
50
25
0
ns
ns
ns
*
ns
N0
N1
N2
CD96+
IFN
F
Response BCG_naive BCG_responsive BCG_unresponsive
Percentage
100
75
50
25
0
ns
ns
ns
ns
ns
N0
N1
N2
IFN
CD96+
G
Condition BCG Naive BCG Exposed
Percentage
100
75
50
25
0
p = 0.592
p = 0.961
p = 0.483
p = 0.967
p = 0.088
N_N0
N_N1
N_N2
N_IFN
N_CD96+
H
Frequency
100%
75%
50%
25%
0%
BCG Naive
BCG Exposed
Sample
Cluster
N0
N1
N2
CD96+
IFN
Chi-Square < 0.05

Figure S7

A

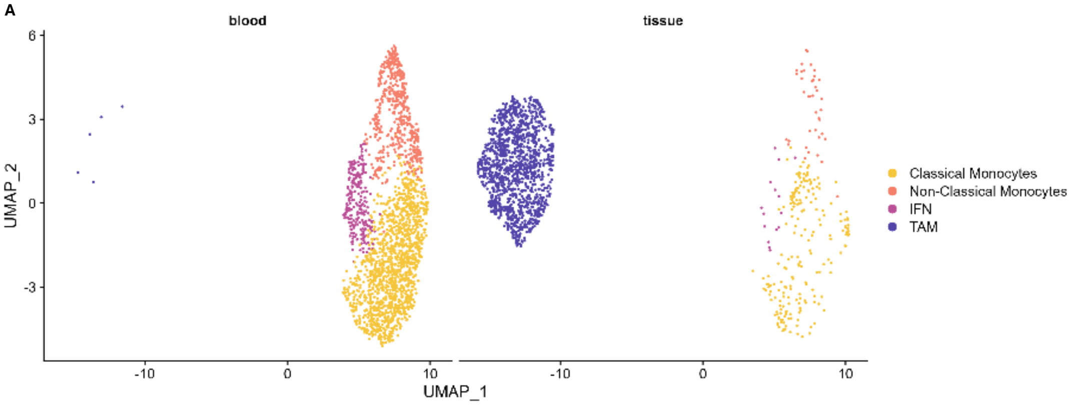

B

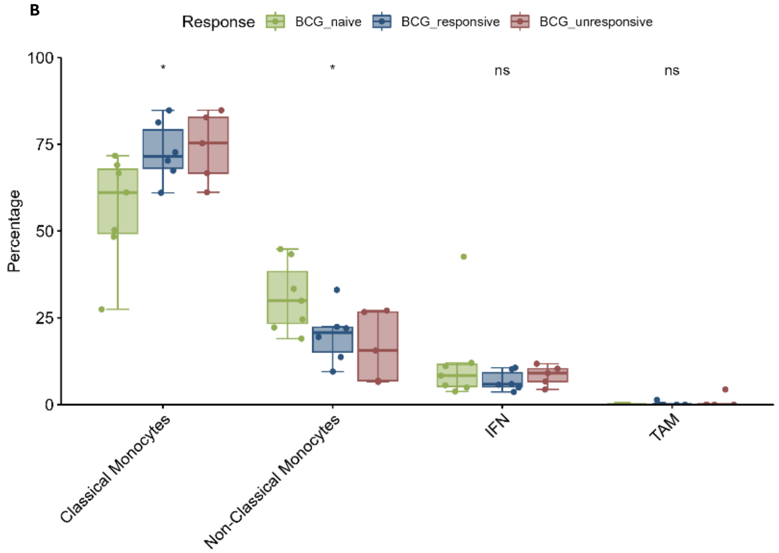

C

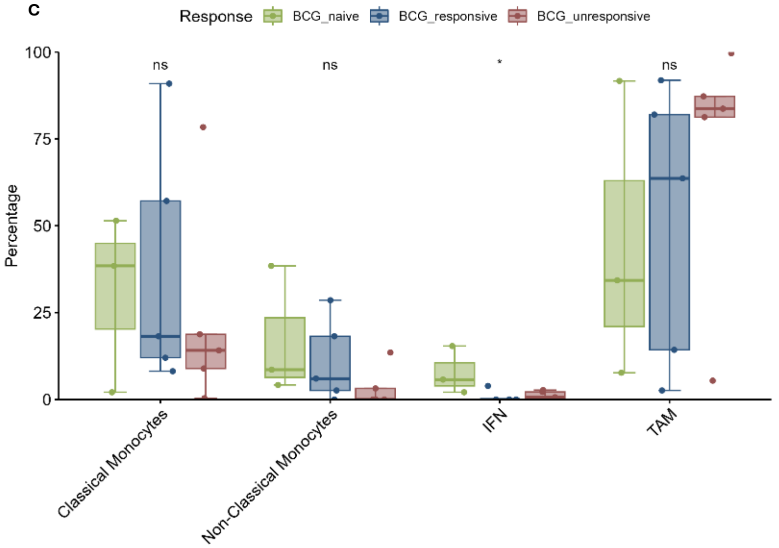

D

Figure S8

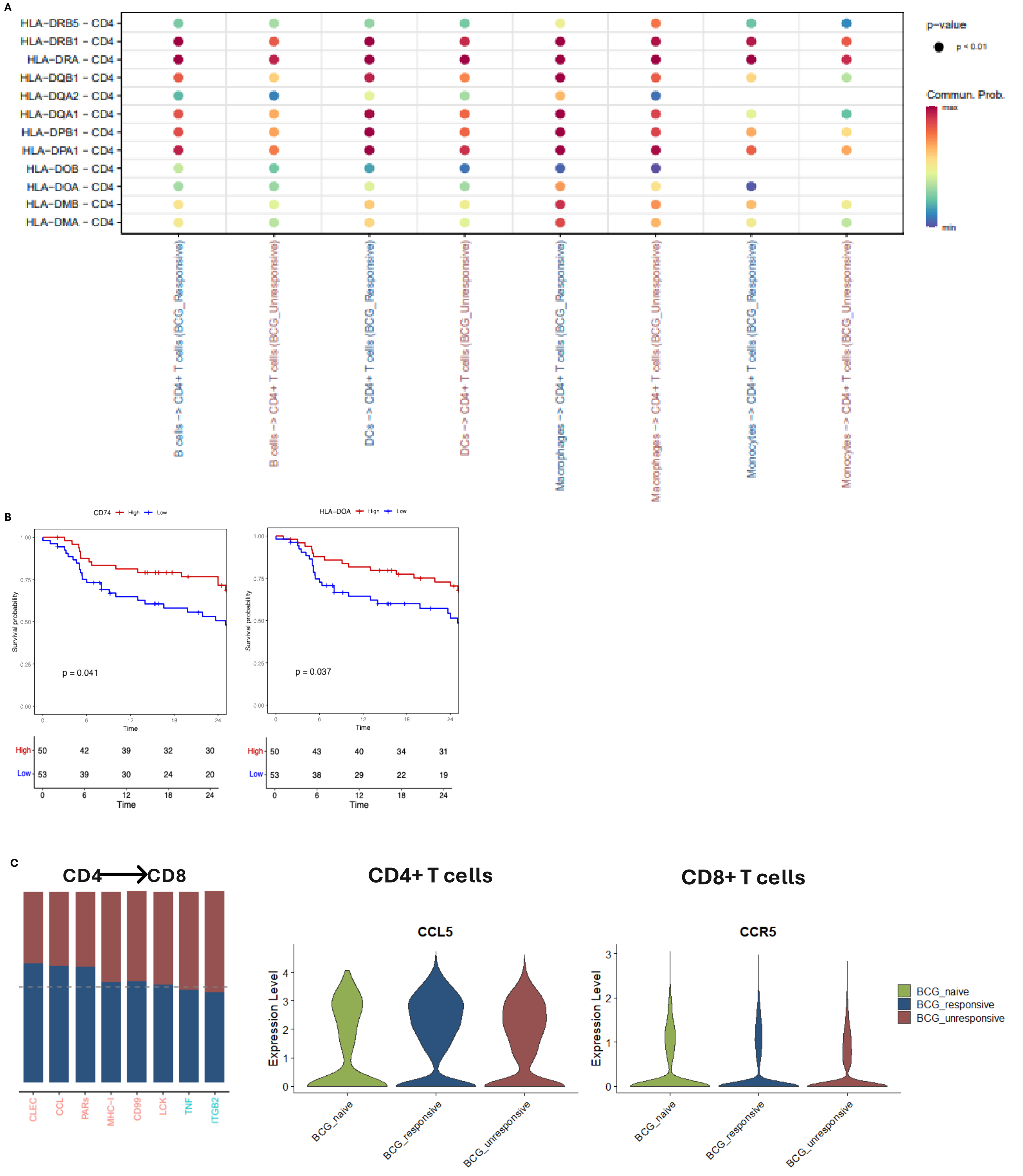

A
HLA-DRB5 - CD4
HLA-DRB1 - CD4
HLA-DRA - CD4
HLA-DQB1 - CD4
HLA-DQA2 - CD4
HLA-DQA1 - CD4
HLA-DPB1 - CD4
HLA-DPA1 - CD4
HLA-DOB - CD4
HLA-DOA - CD4
HLA-DMB - CD4
HLA-DMA - CD4
p-value
p < 0.01
Commun. Prob.
max
min
B cells -> CD4+ T cells (BCG_Responsive)
B cells -> CD4+ T cells (BCG_Unresponsive)
DCs -> CD4+ T cells (BCG_Responsive)
DCs -> CD4+ T cells (BCG_Unresponsive)
Macrophages -> CD4+ T cells (BCG_Responsive)
Macrophages -> CD4+ T cells (BCG_Unresponsive)
Monocytes -> CD4+ T cells (BCG_Responsive)
Monocytes -> CD4+ T cells (BCG_Unresponsive)
B
CD74 High Low
Survival probability
p = 0.041
Time
High 50 42 39 32 30
Low 53 39 30 24 20
HLA-DOA High Low
p = 0.037
High 50 43 40 34 31
Low 53 38 29 22 19
C
CD4 → CD8
CLEC
CCL
PARs
MHC-I
CD99
LCK
TNF
ITGB2
CD4+ T cells
CD8+ T cells
CCL5
CCR5
Expression Level
BCG_naive
BCG_responsive
BCG_unresponsive

Figure S9

A

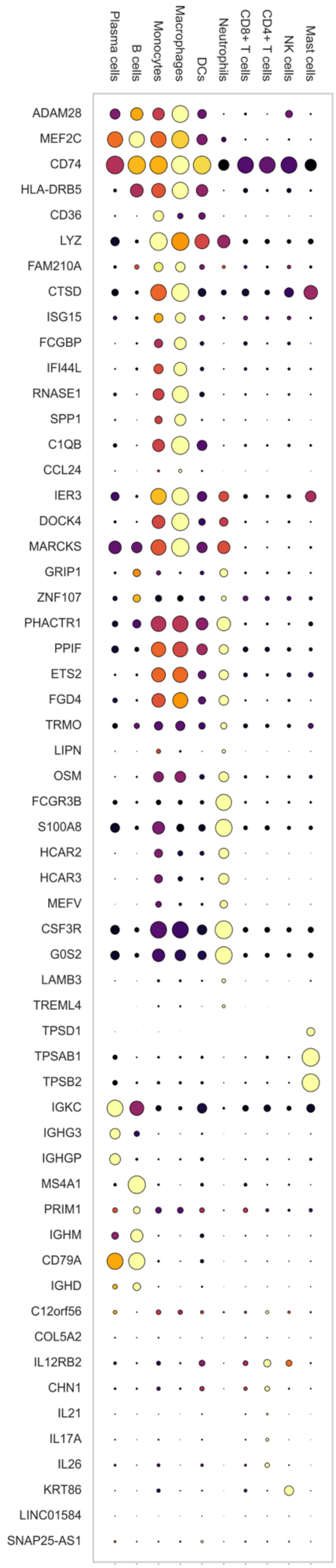
Plasma cells
B cells
Monocytes
Macrophages
DCs
Neutrophils
CD8+ T cells
CD4+ T cells
NK cells
Mast cells
ADAM28
MEF2C
CD74
HLA-DRB5
CD36
LYZ
FAM210A
CTSD
ISG15
FCGBP
IFI44L
RNASE1
SPP1
C1QB
CCL24
IER3
DOCK4
MARCKS
GRIP1
ZNF107
PHACTR1
PPIF
ETS2
FGD4
TRMO
LIPN
OSM
FCGR3B
S100A8
HCAR2
HCAR3
MEFV
CSF3R
G0S2
LAMB3
TREML4
TPSD1
TPSAB1
TPSB2
IGKC
IGHG3
IGHGP
MS4A1
PRIM1
IGHM
CD79A
IGHD
C12orf56
COL5A2
IL12RB2
CHN1
IL21
IL17A
IL26
KRT86
LINC01584
SNAP25-AS1

B

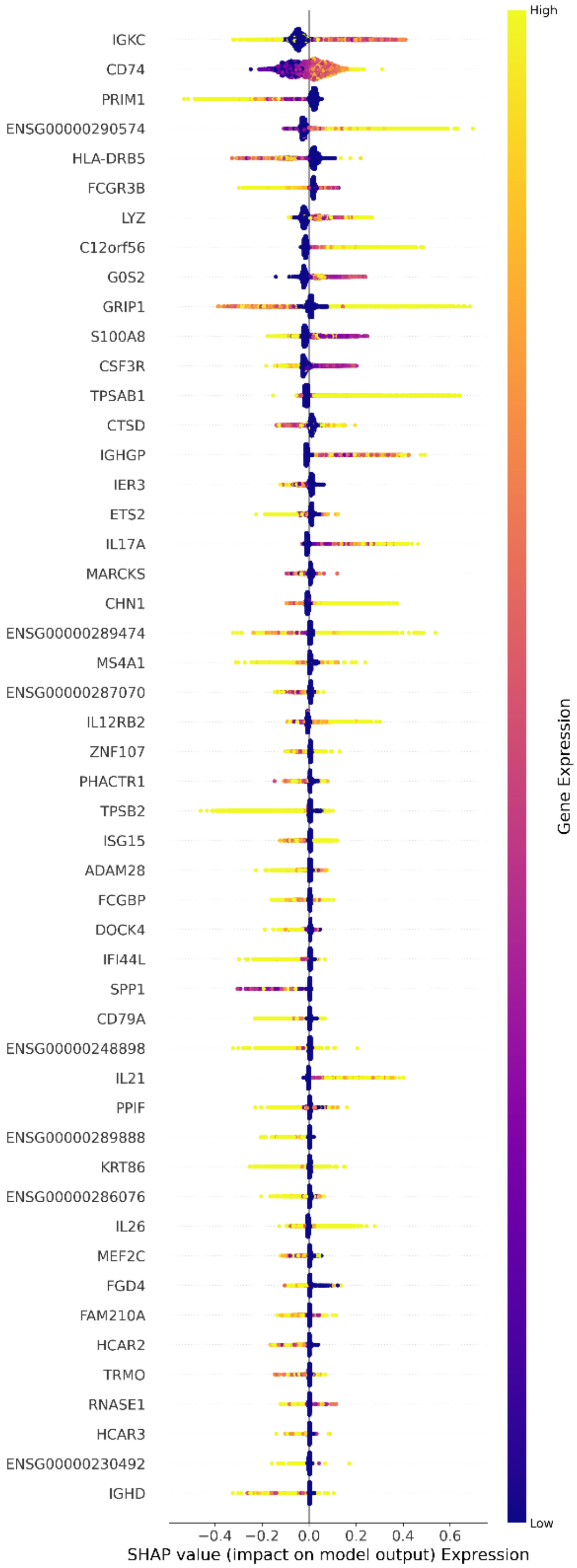
IGKC
CD74
PRIM1
ENSG00000290574
HLA-DRB5
FCGR3B
LYZ
C12orf56
G0S2
GRIP1
S100A8
CSF3R
TPSAB1
CTSD
IGHGP
IER3
ETS2
IL17A
MARCKS
CHN1
ENSG00000289474
MS4A1
ENSG00000287070
IL12RB2
ZNF107
PHACTR1
TPSB2
ISG15
ADAM28
FCGBP
DOCK4
IFI44L
SPP1
CD79A
ENSG00000248898
IL21
PPIF
ENSG00000289888
KRT86
ENSG00000286076
IL26
MEF2C
FGD4
FAM210A
HCAR2
TRMO
RNASE1
HCAR3
ENSG00000230492
IGHD
−0.4
−0.2
0.0
0.2
0.4
0.6
SHAP value (impact on model output) Expression
High
Low
Gene Expression

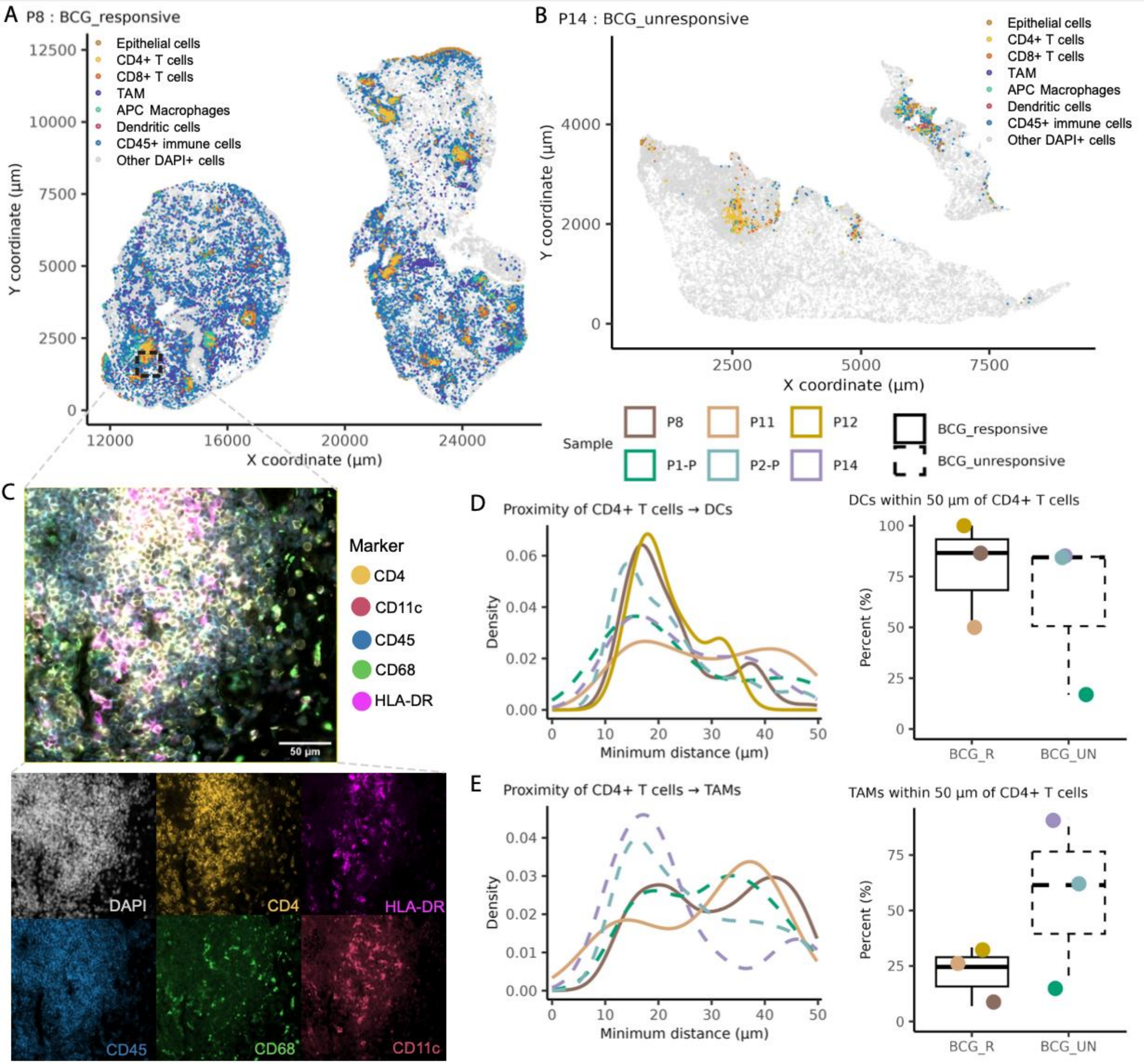
A P8 : BCG_responsive
B P14 : BCG_unresponsive
Epithelial cells
CD4+ T cells
CD8+ T cells
TAM
APC Macrophages
Dendritic cells
CD45+ immune cells
Other DAPI+ cells
Y coordinate (µm)
X coordinate (µm)
Sample
P8
P11
P12
P1-P
P2-P
P14
BCG_responsive
BCG_unresponsive
C
Marker
CD4
CD11c
CD45
CD68
HLA-DR
50 µm
DAPI
CD4
HLA-DR
CD45
CD68
CD11c
D Proximity of CD4+ T cells → DCs
DCs within 50 µm of CD4+ T cells
Density
Minimum distance (µm)
Percent (%)
BCG_R
BCG_UN
E Proximity of CD4+ T cells → TAMs
TAMs within 50 µm of CD4+ T cells
